# The light aging behavior of daylight fluorescent paints: a colorimetric, photographic, Raman spectroscopic and fluorescence spectroscopic study

**DOI:** 10.1186/s40494-022-00812-4

**Published:** 2022-10-27

**Authors:** Lukas Reiß, Thomas Prestel, Sarah Giering

**Affiliations:** 1 Laboratory of Archaeometry, Dresden University of Fine Arts, Güntzstraße 34, 01307 Dresden, Germany; 2 Art Technology, Conservation and Restoration of Painting on Mobile Support, Dresden University of Fine Arts, Güntzstraße 34, 01307 Dresden, Germany

**Keywords:** Daylight fluorescent paints, Fading, Artificial aging, Contemporary art, Colorimetry, DL photography, UVF photography, Raman spectroscopy, Fluorescence spectroscopy, Dye degradation

## Abstract

Daylight fluorescent pigments with their intense color effects have attracted great interest among artists since their market launch in the mid-twentieth century. Since then they have been widely used in the visual arts. The pigments are mainly compositions of organic fluorescent dyes and optical brighteners diluted in an insoluble resin. Due to the susceptibility of the dyes to visible and UV radiation, their lightfastness is comparatively low. This paper presents a comprehensive study of the color and fluorescence changes of daylight fluorescent paints upon exposure in visible light and ultraviolet radiation conducted on mock-ups of commercial daylight fluorescent pigments. The different aging characteristics of the pigments depend on the color tone. They were recorded by means of photographic and colorimetric documentation. In addition, Raman spectroscopy was used to identify the main dyes of the various pigments, even in the complex system of paints, consisting of primer, binder, resin and dyes, and to determine their degradation during aging. Fluorescence spectroscopy revealed that the change in fluorescent color may not only be due to the decrease in dye concentration, but also to the transformation of the original dyes into other fluorescent compounds during light aging. Finally, this paper provides recommendations for the presentation of artworks containing daylight fluorescent pigments.

**Supplementary Information:**

The online version contains supplementary material available at 10.1186/s40494-022-00812-4.

## Introduction

Daylight fluorescent pigments were developed and patented by the Switzer Brothers in 1950 [1]. These fascinating paints possess a high luminosity due to their fluorescence. This fluorescence combined with the reflection achieves a higher intensity in a narrow wavelength range than the spectrum of the ambient lighting, which is why daylight fluorescent paints were first used as eye-catchers by the advertising industry [2]. Artists of various art movements discovered these pigments with their unique color effect and integrated them into their color palettes. Daylight fluorescent paint became particularly important in the artistic work of Rupprecht Geiger, Herbert Aach, Andy Warhol, Frank Stella, Keith Haring and many others [3] and conveyed the artists’ intention. However, the desired presentation is in conflict with conservational concerns. Appropriate handling during storage and exhibition is relevant for the preservation of these works due to their high light sensitivity.

Daylight fluorescent pigments are solid solutions of fluorescent dyes in a polymeric resin. The most commonly used resin is a copolymer of melamine, toluenesulfonamide and paraformaldehyde (MSF resin), as is the case for the pigments investigated in this study. However, resins with a different amine component, polyester, polyamide and polyurethane resins are also used [4, 5]. The fluorescent dyes used for the coloration of the resin are mostly of the xanthene, aminonaphthalimide and benzazolylcoumarin type. As the term “daylight” implies, the majority of these dyes can already be excited to fluorescence by visible light. Common additives are fluorescent brighteners, UV absorbers or antioxidants [5–7]. However, the exact composition is not disclosed by the manufacturers, and although the range of usable colorants is limited, there are considerable differences between the pigments of different manufacturers concerning the exact resin composition, dye selection and mixing ratios. For these reasons, it can be assumed that the aging behavior of pigments from different manufacturers is also different to a certain extent.

A few researchers have taken up the study of the aging of daylight fluorescent paints [8–12]. The paints investigated in these studies were either printing inks on posters [8], ready-to-use products from different manufacturers [9–11], or, as in the present study, paints prepared from the pigments [12]. The extent of the studies varied widely, both in terms of the type of the chosen irradiation and in terms of the measurements made on the aged samples, which included photography [11, 12], color measurements [9, 11], reflectance spectroscopy [8, 9, 11], fluorescence spectroscopy [9, 10, 12] and thin-layer chromatography (TLC) [12]. The influence of the initial pigment concentration on the aging behavior of daylight fluorescent paints, which was already recognized by the artist Herbert Aach [13], was additionally investigated by Yoshizawa and Connors-Rowe et al. [9, 11]. Although the results of the studies varied widely, the main findings were the same: The initial very high reflectance at the emission wavelength initially showed a slight decrease, as evidenced by a darkening of the paint. Subsequently, a blue shift in the emission wavelength was observed, after which this emission also became weaker. Due to dye degradation, the reflectance spectra flattened out, which is equivalent to discoloration of the paint [8, 9, 11]. Depending on the product, UV radiation had mainly an accelerating influence on the fading rate but not on the aging process. However, Connors-Rowe et al. found that some of the constituent dyes are stable to UV radiation, thus the discoloration did not change to white, but to a different color [9]. A high initial pigment concentration resulted in a lower luminosity of the paints before aging due to self-quenching, so that the fluorescent intensity initially increased during aging. In addition, a higher pigment concentration achieved a red shift in the emission band, leading to the assumption that the shift in emission wavelength during aging was due to the decrease in concentration of the dyes [9, 11]. Hinde observed from thin-layer chromatography that the fluorescent dyes contained in daylight fluorescent pigments largely degrade step-by-step in order of decreasing emission wavelength. This means that in the case of red-colored pigments, the red fluorescent dye degraded first, then the yellow fluorescent dye, and so on [12].

The aim of this study is to trace the aging processes by means of different but complementary methods. The results are intended to provide guidance for both analytical identification and conservation practice. The mock-ups produced for the test series presented here contained the daylight fluorescent pigments from Kremer Pigmente in an acrylate binder as the most common binder in commercially available daylight fluorescent paints. The colors were applied over a white primer to simulate the image composition commonly used by artists. Parts of the results of the study conducted here have already been published [14]. Additionally, the results presented here show the influence of UV radiation on the daylight fluorescent pigments from Kremer Pigmente. Further conclusions about dye degradation are drawn from the fluorescence spectra. Raman spectroscopy proves as a useful tool for both dye identification in samples of daylight fluorescent paints and for tracking degradation, even without the use of surface-enhanced Raman spectroscopy (SERS), as highlighted in an article by Campanella et al. [15]. A complete analysis of the dyes contained in daylight fluorescent pigments from Kremer Pigmente using LC–DAD–MS was published very recently by Schmidtke Sobeck et al. [16], which justifies the choice of reference dyes made in the study presented here. The dyes, optical brighteners and non-fluorescent pigments found in their study are summarized in Table 1.

**Table 1:** Dyes, optical brighteners and non-fluorescent pigments found by Schmidtke Sobeck et al. [16]

Pigment	Major components	Minor components
White	Fluorescent Brightener 184	–
Blue	Pigment Blue 15:3 Coumarin 1	–
Green	Pigment Green 7	Solvent Yellow 172 Coumarin 1
Lemon Yellow	Solvent Yellow 172 Fluorescent Brightener 184	–
Golden Orange	Basic Violet 11 Basic Red 1:1 Fluorescent Brightener 184	Sulforhodamine B Solvent Yellow 172
Brick Red	Sulforhodamine B Basic Violet 11 Basic Red 1:1 Solvent Yellow 172 Fluorescent Brightener 184	–
Violet	Sulforhodamine B Basic Violet 11 Unknown violet dye	Basic Red 1:1

## Materials and methods

### Materials and sample preparation

The investigations were carried out on eight daylight fluorescent pigments from Kremer Pigmente. This includes the pigments: White (#56000), Blue (#56050), Green (#56100), Lemon Yellow (#56150), Golden Orange (#56200), Brick Red (#56300), Cyclamen Red (#56400) and Violet (#56450). Plextol D498 from Deffner and Johann (#2557100) was used as a binder for the primer and the paint layer. It is a copolymer based on butyl acrylate and methyl methacrylate with a glass transition temperature of 13 °C and very weak UV-induced visible fluorescence. It corresponds to the common compositions for acrylic binders for artists' paints [17]. Titanium white rutile (#1410000) from Deffner and Johann was used for the primer because of its low fluorescence intensity. The glass slides were obtained from Carl Roth (#0656.1).

The primer was mixed out of rutile, Plextol D498 and water (8 + 8 + 1 parts per volume) with a magnetic stirrer for 15 min, and then applied evenly to the slides in ten very thin layers with a brush. For the color layer, the pigments were mixed with Plextol D498 (1 + 2 parts per volume) for 15 min using a magnetic stirrer. The slightly foamed colors were degassed using a vacuum chamber. A 150 µm thick wet film of the paint was then applied to the primed slides with a squeegee, and the dry film thickness after curing averaged 70 µm. Two mock-ups of each pigment were made, one for aging under daylight LEDs and one for aging under UV LEDs. One mock-up of pure Plextol D498 on the primer was prepared. This sample showed no changes in the spectra during aging.

**Fig. 1:**
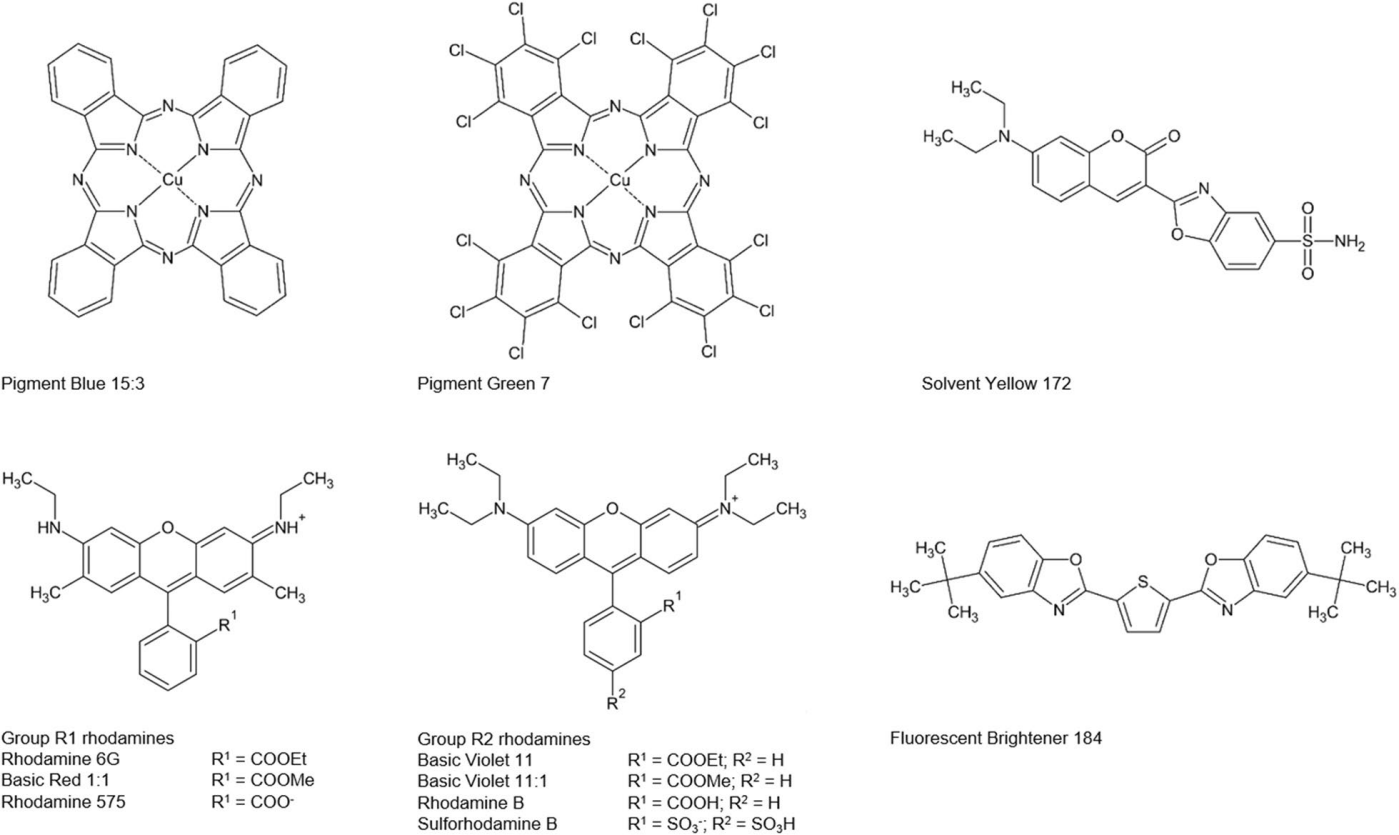
Structures of the pigments, dyes and optical brighteners used as reference materials for Raman spectroscopy; Counter ions for the cationic Rhodamine dyes are not shown

The following pigments, dyes and optical brighteners were used as references: Pigment Blue 15:3 (C.I. 74160) and Pigment Green 7 (C.I. 74260), kindly provided by the Rijkserfgoedlaboratorium in Amsterdam; Sulforhodamine B (#082P1700; C.I. 45100), Basic Violet 11 (#134P0401; C.I. 45175), Basic Violet 11:1 (#124P0400; C.I. 45174) and Solvent Yellow 172 (#045P0408), kindly provided by Neelikon Food Dyes & Chemicals, Ltd.; Rhodamine 6G (#0749.1; C.I. 45160), Rhodamine 575 (#7310.2) and Rhodamine B (#T130.1; C.I. 45170) from Carl Roth; Fluorescent Brightener 184 (#A14928; C.I. 515245) from Alfa Aesar; Basic Red 1:1 (C.I. 45161) from WinChem Industrial Co., Ltd. The structures are given in Fig. 1.

### Accelerated light aging

**Fig. 2:**
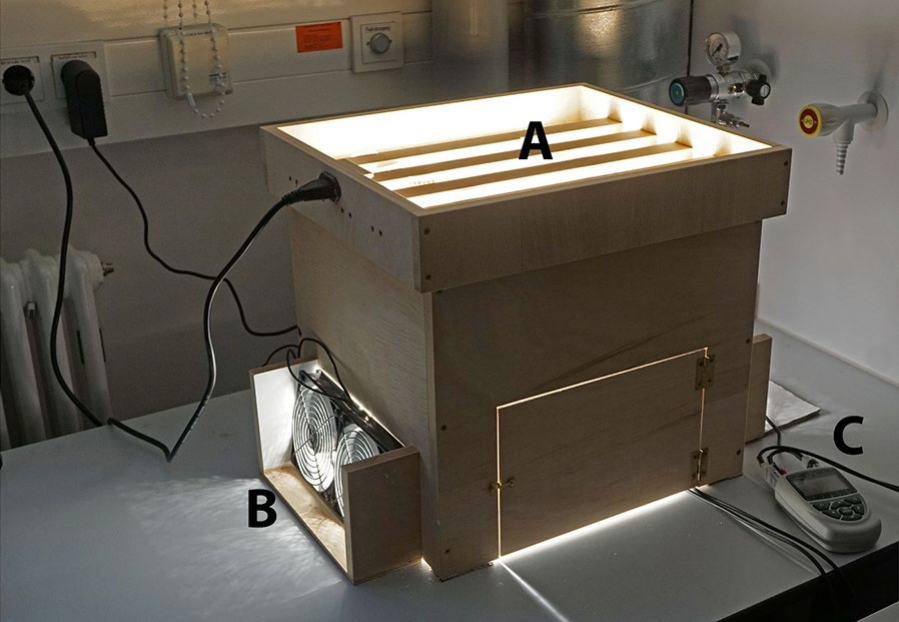
Exposure chamber for light aging experiments; **A** rows for lamps in lid, **B** fans for air ventilation, **C** data logger

Artificial light aging was performed in an in-house constructed exposure chamber (Fig. 2) in which the light sources were mounted on exchangeable lids. For aging under visible light (VIS aging), 20 halogen lamps STAR PAR 16 6.9 W 4000 K CCT from Osram were used and for aging under UV radiation (UV aging), realUV LED Strips (365 nm) from Waveform Lighting were used. The spectra of the lamps and LEDs can be found in the additional supporting files (Additional file 1: Fig. S1). The 5 m strip was cut in pieces of 30 cm length, which were soldered side by side to build a LED plane. The chamber was equipped with fans to keep the temperature at room level. A data logger (Almemo® 2590 from Ahlborn) was used to measure temperature, humidity, illuminance (VIS aging) and irradiance (UV aging) during aging.

The mock-ups were positioned centrally in the exposure chamber, and the positions were swapped regularly to achieve equal exposure for all samples. VIS aging was performed for 181 days, corresponding to an exposure of 360 Mlxh.^1^ This value was converted to the total radiant exposure of 941 kWh/m^2^ within the measured spectral distribution of the LEDs [18]. UV aging was performed for 86 days, while continuously monitoring the power of the UV LEDs. The total radiant exposure was 16.3 kWh/m^2^. For the measurements, the mock-ups were removed from the chamber at specific time intervals.

### Color measurement

Colorimetric values in the CIELab-system were acquired with a KonicaMinolta CM-2600d spectrophotometer. The 3 mm-aperture was used in the mode SCI (specular component included), together with standard illuminant D65 and the 10° standard observer. For each sample and stage in the aging, five single measurements were recorded of which means and standard deviations were calculated. Color differences ΔE00 were calculated according to the CIEDE2000 formula [19].

### Photography

In order to enable the highest possible comparability of the photography, it was necessary to use a clearly defined and unchanging setup. The setup for the visible light photography (DL photography) [20] consisted of the DSLR camera Nikon D500 with a AF-5 Nikkor 16–80 mm lens, daylight flash system Elinchrom Digital 2400 RX with two Digital See Flash Heads and Softboxes. For the color target, a Xrite Colorchecker Classic Mini was used. For the UV-induced visible fluorescence photography (UVF photography) [21], two Hönle UVAHAND 250 UV lamps were used. The camera lens was fitted with a Lee Filters 2C UV cut filter. A non-fluorescent dark gray cardboard was used as the background. Care was taken to use the same equipment, settings and distances for all recordings to ensure reproducibility and comparability.

### Raman spectroscopy

Raman spectroscopic study of the mock-ups was performed on a HORIBA Jobin Yvon XploRA Raman microscope with 50× objective magnification and 785 nm laser. A holographic grating of 600 rulings/mm provided a dispersion of 13 cm^−1^/mm (785 nm). Output laser power was P ≤ 12 mW. NGSLabSpec software was used for measurement. The slides were positioned under the microscope and measured directly. Laser intensity, measurement time, and number of accumulations were adjusted separately for each sample. For Raman spectroscopy, the reference dye powders were pressed in a diamond cell and positioned under the Raman microscope. Due to the fluorescence background, the obtained spectra were baseline corrected for the purpose of visualization using the asymmetric least squares method in the Origin software.

Some Raman bands show a significant variation in intensity between measurements, originating from the acrylate binder Plextol D498 (1733 cm^−1^, 1450 cm^−1^, 1300 cm^−1^, 846 cm^−1^, 813 cm^−1^), the rutile from the primer (608 cm^−1^), impurities of calcite (1085 cm^−1^), and the MSF resin of the daylight fluorescent pigments (1598 cm^−1^, 1377 cm^−1^, 1152 cm^−1^, 1046 cm^−1^, 975 cm^−1^, 795 cm^−1^, 633 cm^−1^) [22]. The variation in intensity is due to different mixing ratios of the color at different locations and slight variation in thickness of the color layer. The spectra were normalized to the band of the resin at 1152 cm^−1^ using the Origin software.

### Fluorescence spectroscopy

Fluorescence spectroscopy was performed on a Varian Cary Eclipse fluorescence spectrometer with the Cary Eclipse Scan software. For spectra acquisition of the aged mock-ups, small samples were cut from the slides, and clamped in the spectrometer's solid sample holder. The sample holder was adjusted to an angle of incidence of 30°. Emission spectra were recorded at an excitation wavelength of 365 nm, and excitation spectra were recorded at the emission wavelength with maximum emission. The fluorescence spectra of the reference dyes are not presented here because a strong red shift occurred due to the formation of J-aggregates, already reported for rhodamine dyes [23, 24].

Since the detector voltage had to be adjusted for each measurement separately, and the sample size varied greatly, the spectra were normalized using the Origin software. Only semi-quantitative conclusions could be drawn from the spectra based on the background intensity and the very weak emission band at 580 nm, which appears in the spectrum of pure Plextol D498 and could only be observed in the spectra of the samples for low dye concentrations. The technical spectra obtained directly from the instrument are shown. These show for the excitation spectra background peaks from the spectral profile of the xenon lamp and for the emission spectra a slight increase at the edges at 400 nm and 700 nm due to the different sensitivity of the detector at different wavelengths [25].

## Results and discussion

**Fig. 3:**
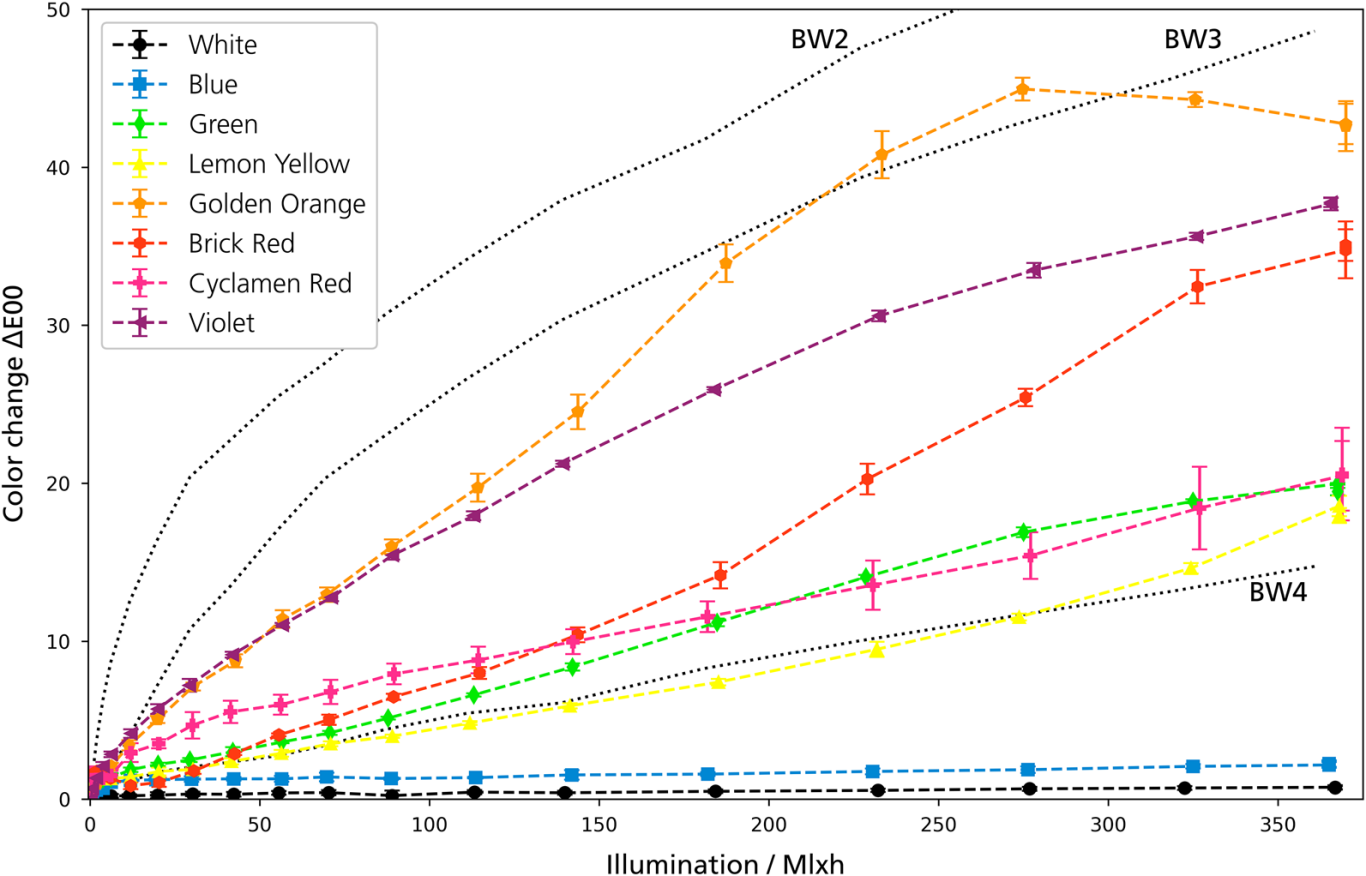
Color Changes ΔE00 of the samples under VIS aging

**Fig. 4:**
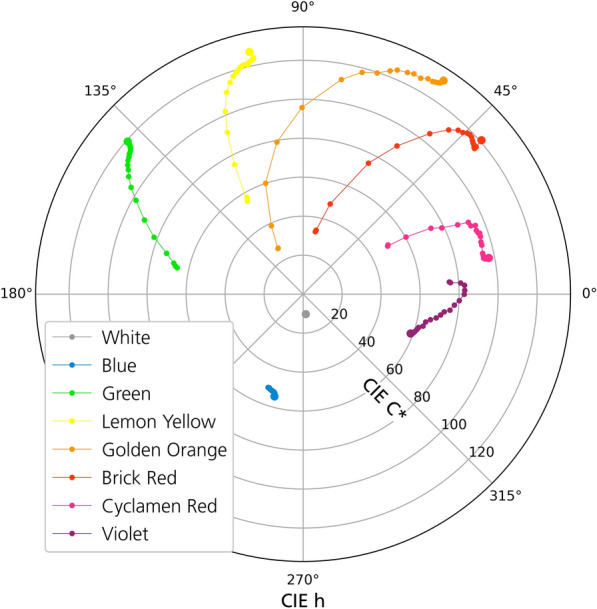
Changes of chroma C* and hue h of the samples under VIS aging; Initial value—indicated by a bigger dot

**Fig. 5:**
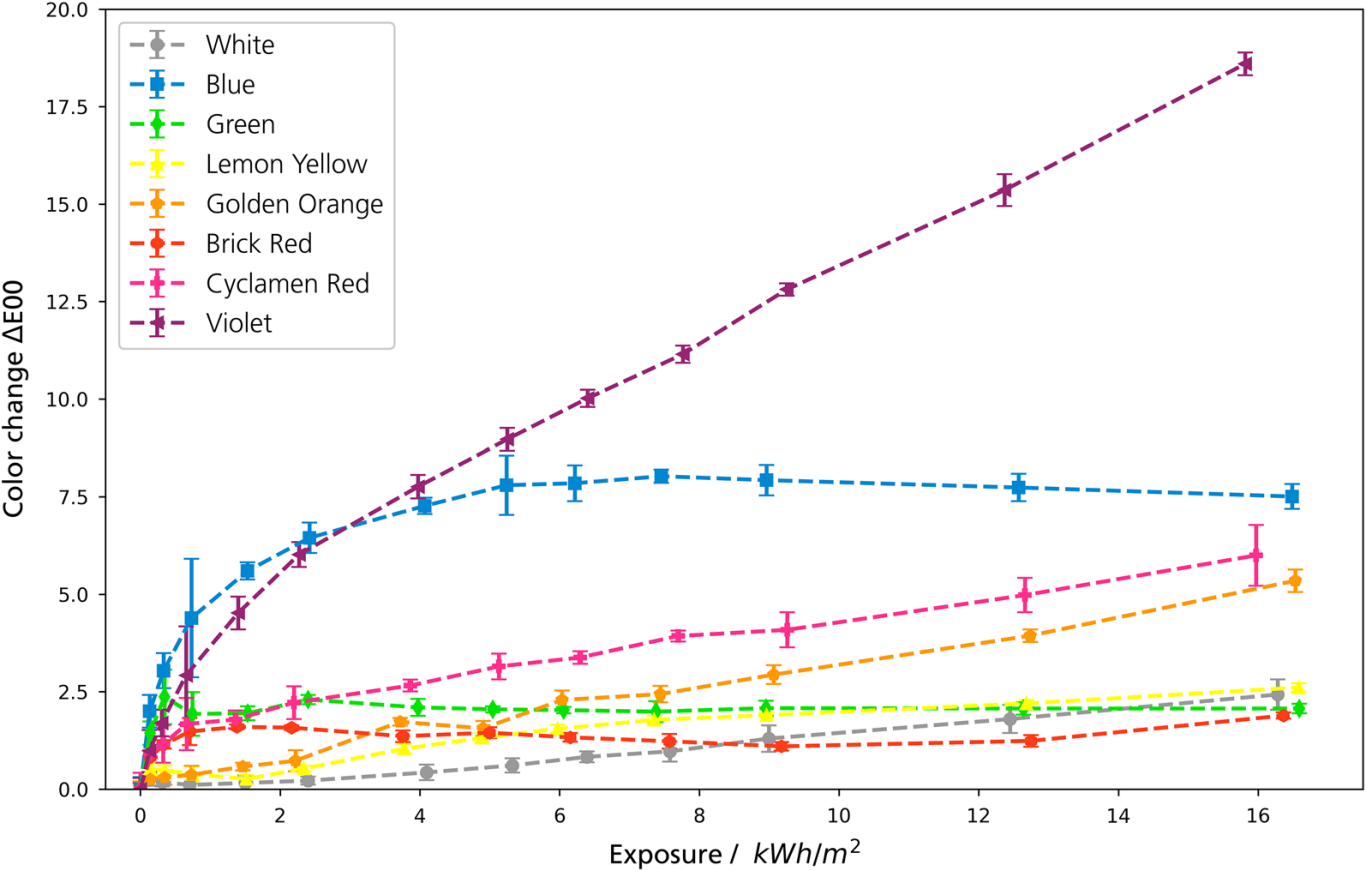
Color Changes ΔE00 of the samples under UV aging

In this section, overviews of all methods for all pigments are shown and briefly explained, followed by detailed information on the individual pigments. Figures 3 and 4 show the changes in color, chroma and hue of the paints under VIS aging. Figure 5 shows the color changes of the paints under UV aging.

The color change of the mock-ups differs strongly. The White and Blue pigments are stable, while the Golden Orange, Brick Red and the Violet pigment show the largest changes due to exposure (Fig. 3). For most samples, a sudden increase of the color change was detected at the beginning of the VIS exposure. In general, the characteristics of the color change of the daylight fluorescent colors were in most cases an increase of the brightness together with a loss of chroma. Exceptions were the Green, Blue and Violet pigments. In VIS aging, the change of the hue was counter-clockwise in the CIE-C*/h-plane for all sensitive mock-ups (Fig. 4). UV aging covered only a small part of VIS aging in terms of energy exposure. However, the initial behavior under UV aging was similar to VIS aging (Fig. 5).

**Fig. 6:**
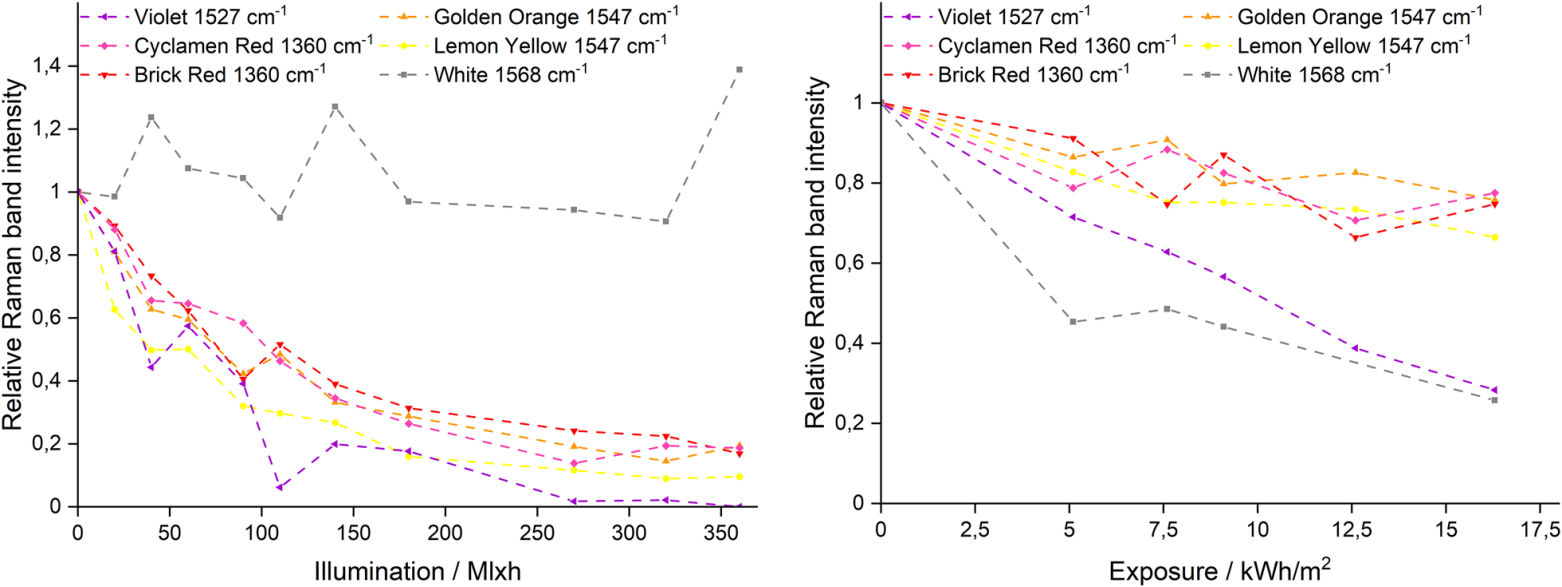
Raman band intensity of the main dyes/brighteners of the pigments during VIS aging (left) and UV aging (right): White—Fluorescent Brightener 184, Lemon Yellow/Golden Orange—Solvent Yellow 172, Brick Red/Cyclamen Red—group R1 rhodamines, Violet—unknown violet dye; Raman spectra of Blue and Green were dominated by the bands of the phthalocyanine pigments PB15:3 and PG7

Figure 6 shows the course of the Raman band intensity of the main dye or the main optical brightener of the individual pigments.

The band intensity of the main dye/brightener decreased exponentially for all pigments except White, whose band intensity remained during VIS aging. In contrast, UV aging, which was significantly shorter than VIS aging in terms of total radiant exposure, showed only a minor decrease in band intensity for most pigments. For the White and the Violet pigment, on the other hand, the band intensity dropped quickly in UV aging.

The emission maxima before and after VIS and UV aging obtained via fluorescence spectroscopy are summarized in Table 2.

**Table 2:** Course of the emission maxima during VIS and UV aging

Pigment	Emission maximum/nm		
	Before aging	VIS aging	UV aging
White	434	440	446
Blue	447	447	447
Green	507	492	507
Lemon Yellow	518	491	518
Golden Orange	580	460	575
Brick Red	600	463	600
Cyclamen Red	602	436	602
Violet	624	605	620

For Blue and White, the emission remained in the blue range at about 440 nm, with the emission of the White pigment shifting somewhat toward higher wavelengths. For the other pigments, the emission wavelength generally shifter to lower wavelengths and UV aging showed a weaker effect on the emission wavelength than VIS aging due to the lower total radiant exposure. The emission wavelength of Green and Lemon Yellow shifted only weakly from the green to the turquoise range, while all red pigments except Violet showed a significant shift from an initial emission wavelength of about 600 nm in the red range to about 450 nm in the blue range. The emission wavelength did not shift continuously, but the intensity of the band at 600 nm decreased, while a second band appeared in the blue range or intermediate in the green range.

The details of the color measurements and photographs are discussed below for the individual pigments and explained with the results of Raman and fluorescence spectroscopy. Spectra that are not included in the main article as well as the Raman spectra of the reference materials (Additional file 1: Fig. S2) can be found in the additional supporting files. A brief summary of the results is given in Table 3.

**Table 3:** Qualitative summary of the color changes during aging with explanation based on spectroscopy results

Pigment	Color changes in VIS aging*	Dye/Brightener degradation
White	No change UV aging: slight decrease in fluorescence	Degradation of Fluorescent Brightener 184 only in UV aging as observed in Raman spectra
Blue	Minimal darkening in color and in fluorescence UV aging: darkening in color and complete loss of fluorescence	Degradation of Coumarin 1 mainly in UV aging
Green	Color Change to turquoise; fluorescence became brighter and then shifted to blue	Degradation of Solvent Yellow 172 Degradation products might fluoresce blue
Lemon Yellow	Became greenish and lost its chroma; fluorescence shifted to blue	Degradation of Solvent Yellow 172 as observed in Raman spectra
Golden Orange	Became yellow and lost its chroma; fluorescence shifted from orange over green to blue	Degradation of group R1 rhodamines followed by the degradation of Solvent Yellow 172
Brick Red	Became orange and lost its chroma; fluorescence shifted from red to blue	Degradation of rhodamines which is directly accompanied by the degradation of Solvent Yellow 172
Cyclamen Red	Became more reddish and lost its chroma; fluorescence shifted from red to blue	Degradation of rhodamines Absence of Solvent Yellow 172 leads to the final fluorescence from Fluorescent Brightener 184
Violet	Became pinkish and the chroma increased at the beginning and finally decreased; fluorescence became much brighter UV aging: rapid color change, characteristics similar to aging in VIS	High dye concentration led to quenching before aging Degradation of an unknown violet dye sensitive to VIS and UV

^*^Due to the lower total radiant exposure in UV aging compared to VIS aging, significantly lower changes occurred in UV aging during the experiment period. Only clear changes in UV aging are listed in the table

**Fig. 7:**
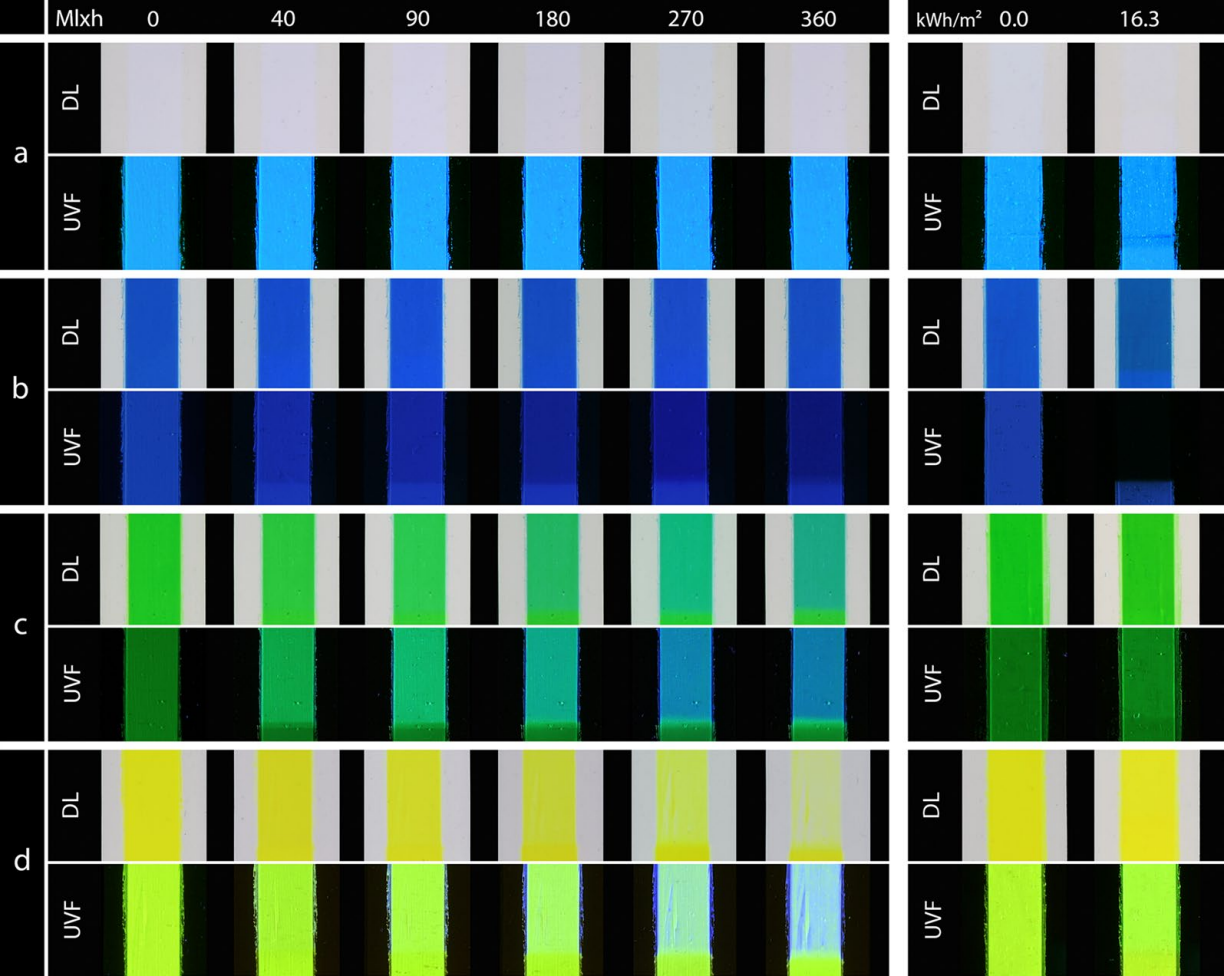
DL (top) and UVF (bottom) photographs of the samples under VIS aging (left) and under UV aging (right); **a** White, **b** Blue, **c** Green, **d** Lemon Yellow

Figure 7 shows the photographs of the mock-ups White, Blue, Green and Lemon Yellow.

### White

The White pigment was stable in VIS exposure. Under UV exposure, a continuous loss in chroma and brightness was detected. The change was evident in a slight decrease in intensity of the fluorescence in the UVF photography of the White sample (Fig. 7a).

**Fig. 8:**
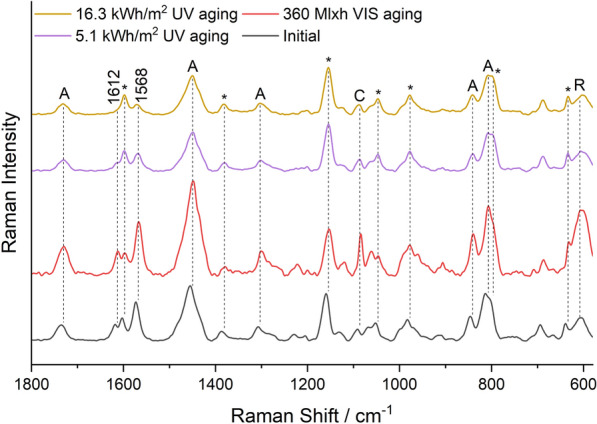
Raman spectra of the White sample before aging, after 360 Mlxh VIS aging and after 5.1 kWh/m^2^ and 16.3 kWh/m^2^ UV aging (from bottom to top); *A* Plextol D498, *R* Rutile, *C* Calcite, *MSF resin

Figure 8 shows the Raman spectra of the White sample. The bands at 1612 cm^−1^ and 1568 cm^−1^ originate from Fluorescent Brightener 184, which is the brightener in most of the daylight fluorescent pigments from Kremer Pigmente [16].

The intensity of these two bands was unaffected in VIS aging, while UV aging significantly reduced it (Fig. 6). This is attributed to the degradation of Fluorescent Brightener 184, which corresponds to a reduction of fluorescence in the UVF photographs. It can be assumed that further UV aging would result in a complete loss of fluorescence. In the fluorescence spectra, the emission wavelength shifted 6 nm from 434 nm for VIS aging and 12 nm for UV aging to higher wavelengths (Additional file 1: Fig. S3). The excitation spectra showed a broad excitation plateau between 340 and 400 nm, which was not influenced by aging.

### Blue

The mock-up of the Blue pigment showed only little change in VIS exposure, which was slightly visible in the DL photography, whereas the fluorescence in the UVF photography decreased significantly. In contrast, UV exposure led to a larger color change at the beginning of the exposure, which was a combination of a loss in brightness and a hue shift in the direction of green. This rapid change was evident in the photographs of the Blue sample (Fig. 7b). The color immediately appeared darker. In particular, the fluorescence decreased rapidly and ultimately disappeared.

For the Blue sample, the Raman spectra are dominated by the bands of the phthalocyanine type Pigment Blue 15:3 (Additional file 1: Fig. S4). Therefore, the degradation of the contained Fluorescent Brightener Coumarin 1 could not be tracked [16]. In the fluorescence spectra, the emission band remained at 447 nm (Additional file 1: Fig. S5). Compared to the excitation plateau of the White sample, the Blue sample showed more of a sharp excitation band.

### Green

In VIS aging, the Green sample appeared darker (decrease of L*) and then more bluish in the DL photography and changed to a turquoise hue (Fig. 7c). The fluorescence in the UV photography initially had a lower intensity than in the other pigments. It became brighter at the beginning of VIS aging and it turned cooler and later bluish. In UV aging, only a small color change was detected at the beginning of the exposure and the fluorescence appeared slightly brighter.

**Fig. 9:**
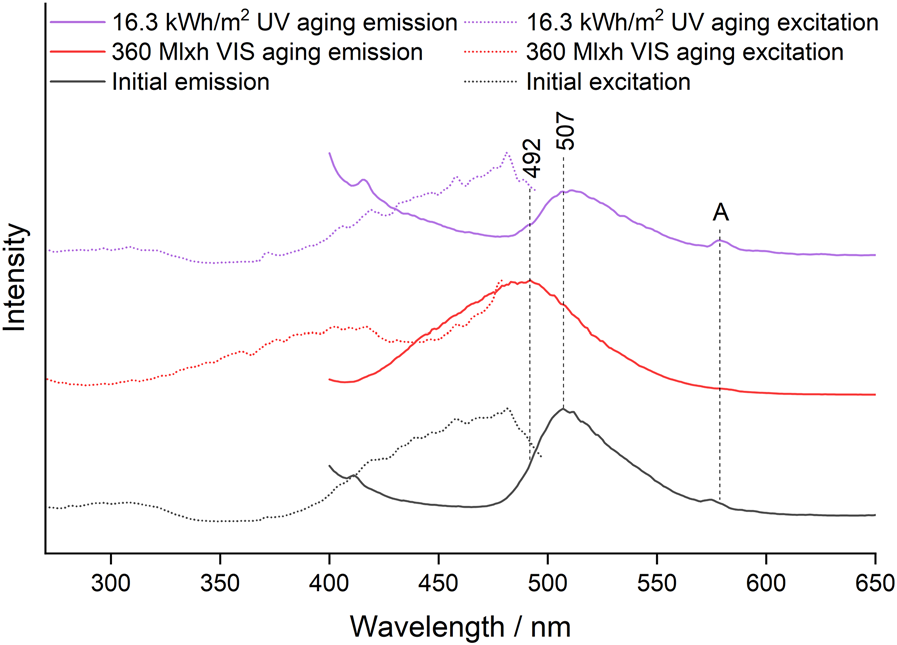
Fluorescence spectra of the Green sample before aging, after 360 Mlxh VIS aging and after 16.3 kWh/m^2^ UV aging (from bottom to top); *A* Plextol D498

For the Green mock-up, the Raman spectra were again dominated by the bands of the phthalocyanine type Pigment Green 7 (Additional file 1: Fig. S6). The fluorescence spectra in Fig. 9 initially showed an emission band at 507 nm from Solvent Yellow 172 [16].

This band showed a blue shift in VIS aging. In the literature, such small changes in the wavelength of the emission band have been attributed exclusively to the reduction of the dye concentration [9, 12]. However, the excitation spectrum showed a completely different shape compared to the state before aging, leading to the assumption that the bluish fluorescence after VIS aging might be due to a degradation product of Solvent Yellow 172. This is in accordance with the loss of yellow hue, leading to the more bluish appearance of the paint in the DL photography (Fig. 7c). Note that Schmidtke Sobeck et al. identified Coumarin 1 as constituent of the Green pigment [16], which could lead to the blue emission under UV. However, in this case the excitation spectrum should convert into the same as for the Blue pigment, which does not happen. The authors performed own TLC and HPLC–MS/MS measurements on the daylight fluorescent pigments from Kremer Pigmente and were unable to detect Coumarin 1 on the TLC plate and only in very small traces in the HPLC–MS/MS. A TLC plate can be found in the additional supporting files (Additional file 1: Fig. S7). As for UV aging, no blue shift of the emission at 507 nm was detected, indicating that there was little degradation of Solvent Yellow 172.

### Lemon Yellow

The Lemon Yellow paint showed a continuous color change in VIS aging. The main contribution was a decrease of the chroma and a change to a more greenish hue. The brightness remained at high L*-values. Only at the beginning of exposure, the yellow appeared slightly darker (Fig. 7d). The fluorescence shifted from strong yellow-green to a cooler green until it appeared bluish. In UV exposure, a linear increase of the color change was found (Fig. 5). No change was visible in the DL photographs, but the fluorescence shifted slightly to a brighter hue.

**Fig. 10:**
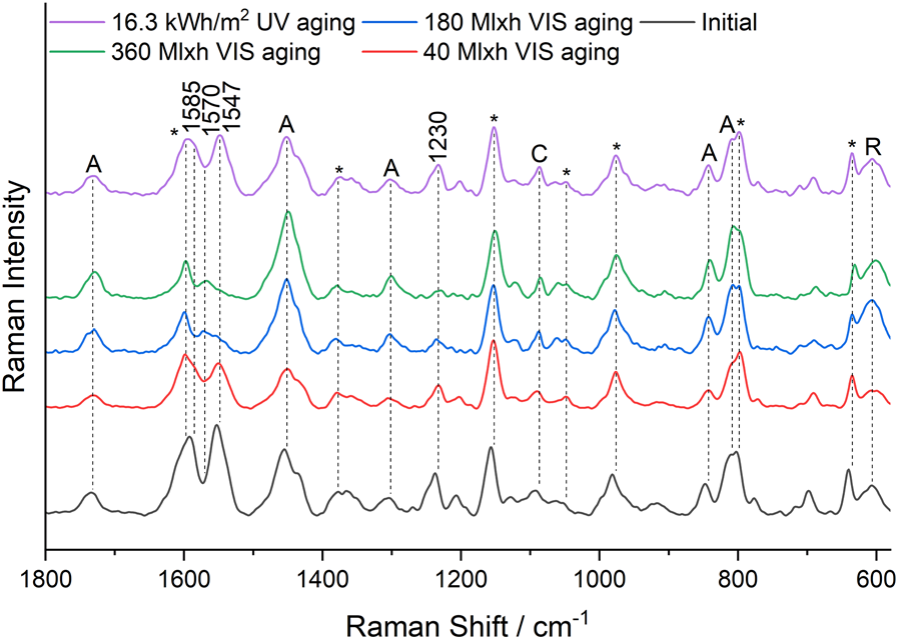
Raman spectra of the Lemon Yellow sample before aging, after 40 Mlxh, 180 Mlxh and 360 Mlxh VIS aging and after 16.3 kWh/m^2^ UV aging (from bottom to top); *A* Plextol D498, *R* Rutile, *C* Calcite, *MSF resin

The Raman spectra of the Lemon Yellow sample in Fig. 10 initially showed the intense bands of Solvent Yellow 172 at 1585 cm^−1^, 1547 cm^−1^, and 1230 cm^−1^.

These bands rapidly decreased in VIS aging (Fig. 6), revealing the band of Fluorescent Brightener 184 at 1570 cm^−1^, also reported in the literature for this pigment [16]. UV aging resulted in the reduction of the band intensity of Solvent Yellow 172 as compared to the band of the resin at 1152 cm^−1^ as well. In the fluorescence spectra (Additional file 1: Fig. S8), the emission band shifted from 518 nm in the green region to 491 nm in VIS aging and the excitation curve showed the same feature at 420 nm as in the Green sample (Fig. 9). Compared to the Green pigment, the Lemon Yellow pigment contains Fluorescent Brightener 184 that could be accountable for the blue fluorescence. However, the emission maximum did not shift until that of the White sample at 434 nm. This again underlines the assumption that the degradation product of Solvent Yellow 172 is responsible for the blue fluorescence.

**Fig. 11:**
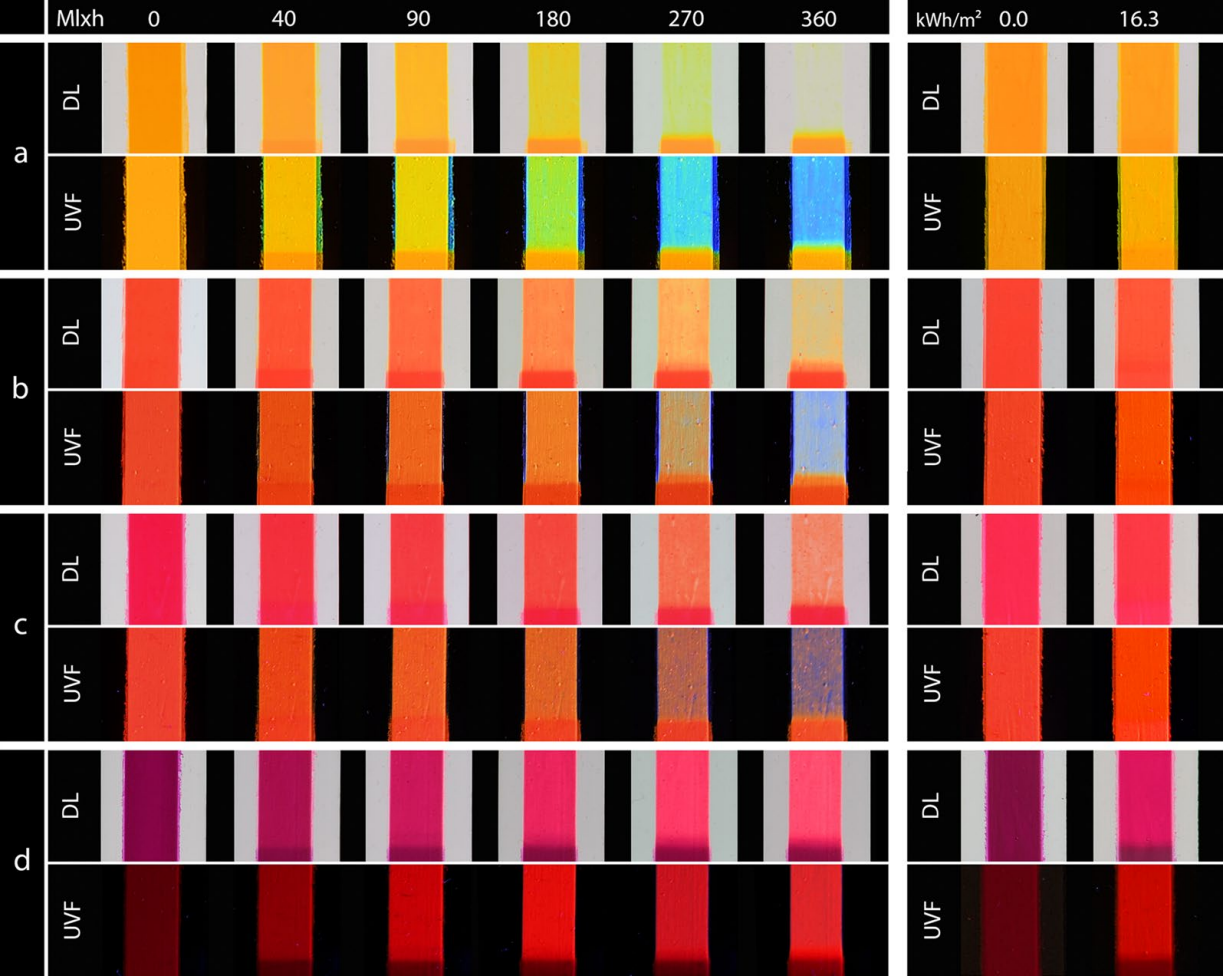
DL (top) and UVF (bottom) photographs of the samples under VIS aging (left) and under UV aging (right); **a** Golden Orange, **b** Brick Red, **c** Cyclamen Red, **d** Violet

The photographs of the second set of mock-ups are presented in Fig. 11 (Golden Orange, Brick Red, Cyclamen Red and Violet). Since Golden Orange and Brick Red, as well as Cyclamen Red and Violet showed a somewhat similar aging behavior, the following evaluations are summarized.

### Golden Orange and Brick Red

Golden Orange and Brick Red showed similar fading curves (Figs. 3, 4), even though the initial values were different and Golden Orange faded faster in VIS aging than Brick Red. The color change was dominated by a change in hue and an increase of the brightness. In later stages of aging, the change in hue was so high that there was an overlap with the neighboring color, meaning that the Brick Red sample took on the hue of the unaged Golden Orange sample in VIS aging. The same applied to the aged Golden Orange and the unaged Lemon Yellow. In the photographic documentation, these colors appeared lighter after a short time and faded to an almost colorless state in VIS aging (Fig. 11a and b). The fluorescence of the Golden Orange sample in UVF photography gradually changed from bright orange to yellow to green to blue, while the fluorescence of the Brick Red sample shifted from red to orange and then revealed blue fluorescence during the decay of the orange fluorescence. Like in VIS aging the Golden Orange sample appeared to be more affected by UV aging during the test period than the Brick Red sample. After UV aging, the Golden Orange color appeared lighter and slightly more transparent in daylight. The fluorescence appeared slightly cooler and more yellowish than before aging. Brick Red appeared slightly lighter in the DL and UVF photographs after UV aging.

**Fig. 12:**
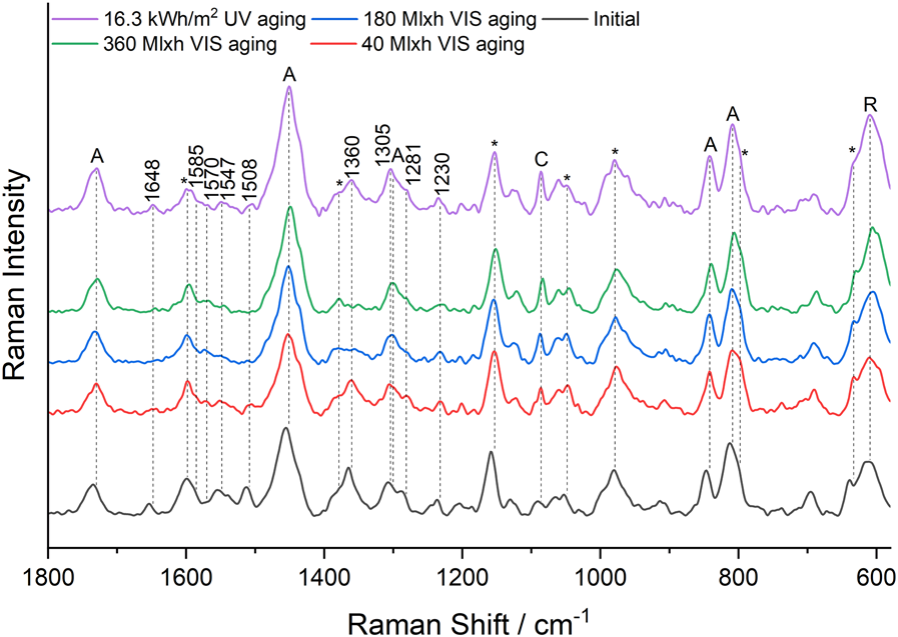
Raman spectra of the Brick Red sample before aging, after 40 Mlxh, 180 Mlxh and 360 Mlxh VIS aging and after 16.3 kWh/m^2^ UV aging (from bottom to top); *A* Plextol D498, *R* Rutile, *C* Calcite, *MSF resin

Figure 12 shows the Raman spectra of the Brick Red mock-up. They show multiple additional bands of rhodamine dyes, which can be divided into two groups according to the substitution of the xanthene skeleton (Fig. 1).

**Fig. 13:**
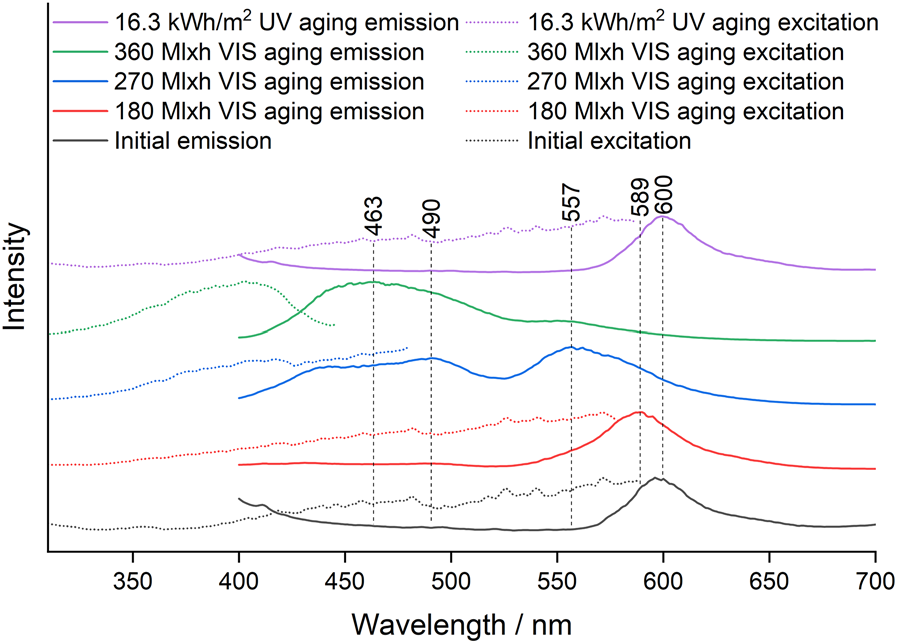
Fluorescence spectra of the Brick Red sample before aging, after 180 Mlxh, 270 Mlxh and 360 Mlxh VIS aging and after 16.3 kWh/m^2^ UV aging (from bottom to top)

Group R1 consists of Rhodamine 6G, Basic Red 1:1 and Rhodamine 575, and shows intense Raman bands at 1518–1508 cm^−1^, 1360 cm^−1^, 1308–1314 cm^−1^ and a weak band at 1648 cm^−1^ (Additional file 1: Fig. S2). Group R2 contains Rhodamine B, Sulforhodamine B, Basic Violet 11 and Basic Violet 11:1, and shows a significantly more intense Raman band at 1648 cm^−1^ than group R1, as well as other intense bands at 1527 cm^−1^, 1507 cm^−1^, 1355 cm^−1^ and 1277 cm^−1^. Brick Red contains both group R1 and R2 rhodamines [16], and the bands of both were degraded in VIS aging (Fig. 6). In addition, the bands of Solvent Yellow 172 (1585 cm^−1^, 1547 cm^−1^, and 1230 cm^−1^), which showed low intensity due to the low concentration compared to the Lemon Yellow sample, disappeared upon VIS aging. In UV aging, the band intensity decreased only negligibly during the period of exposure (Fig. 12). Despite the mixture of dyes in the Brick Red pigment, the corresponding fluorescence spectra in Fig. 13 show only one emission band of the rhodamines at 600 nm before aging.

This is probably explained by Förster resonance energy transfer (FRET), which is addressed in the discussion section. At the beginning of VIS aging, there was a slight shift of the emission band to lower wavelengths. As aging progressed, the concentration of rhodamines decreased as far as the average distance between the donor molecules of Solvent Yellow 172 and the rhodamine molecules increased far enough that FRET no longer occurred sufficiently. As a result, a second broad emission plateau formed, consisting of the emission band of Solvent Yellow 172 and Fluorescent Brightener 184, explaining the observed gradual loss of orange fluorescence and the increase in blue fluorescence after 270 Mlxh VIS aging in Fig. 11b. As the degradation of rhodamines and Solvent Yellow 172 progressed, the emission shifted further into the blue range.

Based on the Raman and fluorescence spectroscopic results the Golden Orange sample behaved like a mixture of the Brick Red sample and the Lemon Yellow sample (Additional file 1: Figs. S9 and S10). This means that the Raman band intensity of Solvent Yellow 172 and thus its concentration were significantly higher than in the Brick Red sample before aging and only group R1 rhodamines could be found. The development of the emission maxima was similar to that of Brick Red, with the emission band initially at 580 nm because group R1 rhodamines have an orange fluorescence compared to the red fluorescence at about 600 nm of group R2 rhodamines. At the intermediate stage, a plateau with two emission maxima at the lower wavelengths did not form as for Brick Red, but a second band formed at 501 nm due to the higher concentration of Solvent Yellow 172. This explains the yellow-green fluorescence of the Golden Orange mock-up after 180 Mlxh VIS aging as a result of the decreasing fluorescence of the rhodamines and the appearance of the green fluorescence of Solvent Yellow 172 (Fig. 11a).

### Cyclamen Red and Violet

Cyclamen Red and Violet showed an increase in brightness due to VIS aging (Fig. 11c and d). Cyclamen Red changed its hue to a more reddish color and lost its chroma (Fig. 4). The color change of Violet was unique to this pigment, the chroma increased through the VIS exposure and decreased again at the end of the aging (Fig. 4). At this fading stage, the color became similar to the unaged Cyclamen Red. The DL photography during VIS aging shows that the Cyclamen Red sample initially appeared warmer after a short aging period (Fig. 11c). As aging progressed, the color became lighter and warmer until it was almost transparent. A change of the fluorescence in the UVF photography became perceptible after 90 Mlxh. In the course of VIS aging, the fluorescence first became lighter and then more bluish. On the other hand, after a short aging time, the Violet sample appeared much lighter and took on a pinkish color (Fig. 11d). At a later stage of VIS aging, it became more transparent. The originally weaker fluorescence became more intense during degradation and changed to a bright red. UV aging showed the same aging characteristics as VIS aging for both pigments. However, Violet faded much faster in UV aging compared to all the other pigments.

**Fig. 14:**
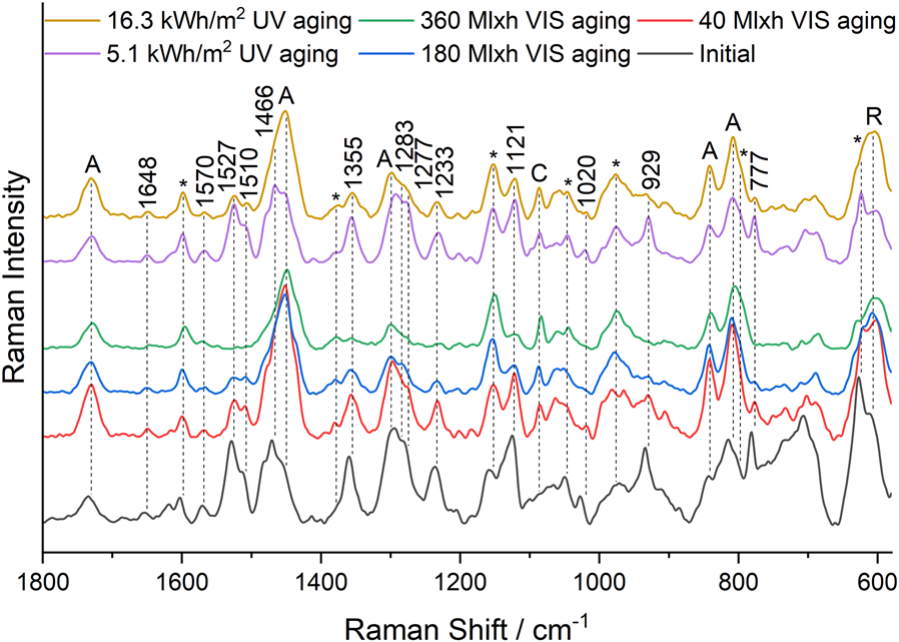
Raman spectra of the Violet sample before aging, after 40 Mlxh, 180 Mlxh and 360 Mlxh VIS aging and after 5.1 kWh/m^2^ and 16.3 kWh/m^2^ UV aging (from bottom to top); *A* Plextol D498, *R* Rutile, *C* Calcite, *MSF resin

For all the previously mentioned red-colored pigments, the bands of the rhodamines in the Raman spectra showed a much lower intensity than the main band of the resin at 1152 cm^−1^ even before aging, indicating a comparatively low concentration of the dyes in the pigments. In contrast, the Raman spectrum of the Violet sample in Fig. 14 shows very intense bands of an additional violet dye at 1570 cm^−1^, 1527 cm^−1^, 1510 cm^−1^, 1466 cm^−1^ with a shoulder at 1475 cm^−1^, 1233 cm^−1^, 1121 cm^−1^, 1020 cm^−1^, 929 cm^−1^ and 777 cm^−1^.

**Fig. 15:**
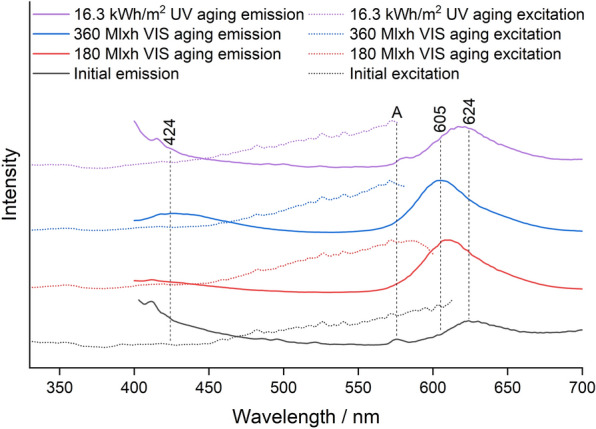
Fluorescence spectra of the Violet sample before aging, after 180 Mlxh and 360 Mlxh VIS aging and after 16.3 kWh/m^2^ UV aging (from bottom to top); *A* Plextol D498

Although the identity of this dye could not be clarified by Raman spectroscopy due to lack of reference substances, it can be considered as the cause of the different and faster aging behavior of the Violet sample compared to the other samples, since the bands are degraded in both VIS and UV aging (Fig. 6). Therefore, unlike the other red pigments, the Violet sample showed a change from violet to a hue similar to the Cyclamen Red sample in both VIS aging and UV aging. However, the fluorescence spectra in Fig. 15 show that there was no change in emission wavelength in UV aging, which means that the unknown dye does not fluoresce.

The high dye content in the Violet pigment probably led to quenching before aging, which accounts for the weak fluorescence of the Violet sample before aging as well as its initial increase during aging. In VIS aging, the behavior of the emission band was the same as for the other red pigments. However, due to the absence of Solvent Yellow 172 in the Violet pigment, no emission band in the green region occurred in the meantime, and the blue emission of Fluorescent Brightener 184 at the end of VIS aging was at 424 nm, in the range of the emission of the White pigment. The same applied for the spectra of the Cyclamen Red sample, which also does not contain Solvent Yellow 172 (Additional file 1: Figs. S11 and S12). Compared with the other red pigments, the absence of Solvent Yellow 172 in Cyclamen Red and Violet also meant that the excitation at 450 nm was still relatively weak, whereas in the excitation spectra of the other red pigments there was already a clear fluorescence excitation at 450 nm.

## Discussion

The study in this paper represents the central light aging phenomena of daylight fluorescent pigments for practical use, as the prepared mock-ups replicate a common application technique. Artists usually apply these pigments pure in a binder onto a white primer, as this creates the highest luminosity. However, the aging behavior of the daylight fluorescent pigments in other binders, in mixtures with other pigments and on other primers can only be derived to a limited extent from this study. The rutile pigment in the primer, for example, has a strong reflection in the visible light range and could thus lead to higher effective exposure in the pigment layer compared to mock-ups applied without primer. This appears especially obvious for thinner paint layers, as can be seen at the edges of the paint layers in Figs. 7 and 11, but only for VIS aging, since rutile absorbs UV radiation to the greatest extent possible [26].

The fading of daylight fluorescent colors is already stimulated by visible light without spectral parts in UV. Depending on the dye composition, the different pigments show different color changes. For most colors, the hue of the fluorescence shifts to shorter wavelengths into the bluish range. On the other hand, the blue fluorescent color of the Blue paint merely becomes weaker because the containing Coumarin 1 seems to be somewhat susceptible to visible light and very susceptible to UV radiation. Due to the strong color change, some color tones approximate each other. There is a risk that hues will be misinterpreted in practice on the object. For example, an aged violet appears like a pink.

The UV aging in this study was significantly lower than VIS aging in terms of energy exposure because the UV LEDs had lower power than the VIS LEDs. Nevertheless, based on the details from the color measurements, it can be assumed that the aging behavior in UV is approximately the same as in VIS for all pigments except White, Blue and Violet. These have already changed faster under UV aging than VIS aging due to the high susceptibility of the optical brighteners and the unknown violet dye to UV radiation, as confirmed by Raman spectroscopy. However, due to the susceptibility of the optical brighteners to UV radiation, it can be assumed that prolonged UV aging would also cause the blue fluorescence to fade in the other pigments, especially since the concentration of Fluorescent Brightener 184 in these pigments is much lower than in the White pigment. This prediction contrasts with the results obtained by Connors-Rowe et al. [9] who generally found faster aging in both VIS and UV, possibly due to a different paint layer thickness and the fact that their image carrier was paper containing optical brighteners. The fluorescence of these optical brighteners could lead to increased excitation of the dyes in the paint layer, which are consequently degraded more quickly. In addition, the green fluorescent dye contained in the Dr. Ph. Martin’s products they examined appeared to be stable to UV, which cannot be assumed for Solvent Yellow 172 in the pigments from Kremer Pigmente. It can also be anticipated that the pigments from DayGlo Color Corp. behave differently in the comparison of VIS aging and UV aging than those from Kremer Pigmente, since Schmidtke Sobeck et al. could only detect Coumarin 1 in the Horizon Blue pigment from DayGlo Color Corp. but no other optical brightener in the other pigments [16].

The VIS aging showed that the mock-ups have a light sensitivity similar to BW3–BW4, which is a very sensitive color in terms of museum lighting [27]. The exposure of 360 Mlxh corresponds to a museum presentation for 600 years at an illumination of 200 lx (assuming 3000 opening hours per year). Thus, this study simulates the complete lifetime of such colors in terms of light aging. Other lightfastness experiments using Microfading Tester on similar daylight fluorescent colors have found a sensitivity comparable to BW2–3, but only on a very small exposure compared to the exposure in the present study [28, 29]. However, for conservation purposes, the following question is important: when does a color change become visible? This question can be answered by looking at the exposure after which a so-called Just Noticeable Difference of ΔE00 = 1.5 occurred [30]. For all colors (except Blue and White, which were stable in VIS aging), this color change occurred between 2–6 Mlxh, which also can be observed in the photographs at the stage of 40 Mlxh (Figs. 7 and 11). In museums, this exposure corresponds to a 3–10 years permanent exhibition, a period that illustrates the high light sensitivity of these pigments.

Since the changes in the pigments already occur in visible light and after a short-time exposure, permanent exhibition of the objects is strongly discouraged from a conservational point of view. Even the use of low-UV lighting is not sufficient here to slow down the aging process significantly. UV, black light, and other light sources with a high amount of short wavelength radiation should only be integrated into the exhibition concept with caution. To preserve objects, the annual exposure should be limited to a defined maximum.^2^ If an artwork is intended for presentation with higher light exposure or black light, the authors recommend that a concept for dealing with light damage be discussed with the artist, if possible. If not exhibited, it is imperative that these objects are stored under complete exclusion of light. Monitoring via photography and colorimetry facilitates the ability to determine changes in the artworks.

For the photographic documentation in this study, it was important that the photographs are as much comparable to each other as possible. This was achieved by exactly reproducing the set-up and using a standardization method with color targets. The comparability of different UVF photographs can be increased through standardization according to the UV-Innovations system with the UV-Gray and the Target-UV.^3^ This method appears to be suitable for use with daylight fluorescent pigments, even if the reproduced color impression differs from the perceived fluorescence, particularly with regard to the blue hues. This is mainly due to the white balance and the UV cut filter, which absorbs a small part of the visible blue spectrum. The photographs provide visual information about the actual color of the samples and their fluorescence. However, it has to be considered that the appearance of the photographs is strongly dependent on the camera settings and the image processing, as well as on the screen display or print reproduction used. Therefore, photographs can only be used comparatively for other purposes to a limited extent.

Despite the complexity of the Raman spectra with the bands of the primer, binder, resin and dyes, some dyes and optical brighteners could be identified directly in the Raman spectra, and the degradation of the main dyes could be detected in most pigments. It was found that the dyes are mainly degraded under visible light, while the optical brighteners are only susceptible to UV radiation. In fluorescence spectroscopy, the emission spectra show only one emission band despite the mixture of dyes. This is explained by FRET, which results in non-radiative excitation of an acceptor dye in the presence of an excited donor dye whose emission wavelengths overlap with the excitation spectra of the acceptor dye, enhancing the fluorescence of the acceptor dye and quenching that of the donor dye [31, 32]. This thesis is supported by the following estimation of the average distance between the brightener/dye molecules, which is based on some assumptions:

- The density ρ of the daylight fluorescent pigments is about 1 g/cm^3^, since they are slightly lighter than water.
- The total mass fraction ω of optical brighteners and dyes is 1%, as indicated in the literature as the usual concentration range [7].
- A uniform distribution and an equal size of optical brighteners and dyes in the resin is assumed.
- The molar mass M is fixed at 400 g/mol. Although this varies between 231 g/mol for Coumarin 1 and 559 g/mol for Sulforhodamine B, these values also lead to the same conclusion in the calculation.

Under these assumptions, the particle density n is 1.51·10^19^ particles/cm^3^ according to the following equation:

$$n=\frac{\rho \cdot \omega \cdot {N}_{A}}{M}=1.51\cdot {10}^{19}\,{ \text{cm}}^{-3}$$

Thus, a uniform distribution and the assumption of equal particle size result in an average distance d of 4.05 nm:

$$d=\sqrt[3]{\frac{1}{n}}=4.05\cdot {{10}^{-7}}\, \text{cm}$$

This is within the range of typical donor–acceptor pair-dependent Förster distances of 1.5 nm to 6 nm at which FRET occurs [25]. Additional measurements on the spectral overlap of potential FRET pairs in the daylight fluorescent pigments of DayGlo Color Corp., Radiant Color and Kremer Pigmente have already been performed [29, 31]. With respect to fluorescence spectroscopy, it has been previously stated in the literature that minor blue shifts of the emission band are due to the reduction of the concentration of the original dye [9, 12]. However, this study shows that the wavelength of the emission band of the pigments containing Solvent Yellow 172 was significantly higher after VIS aging as compared to the pigments without that dye. The excitation spectrum also changed its shape significantly in these cases. This leads to the assumption that the reduction of the emission wavelength additionally results from the conversion of Solvent Yellow 172 into another substance fluorescing in the blue range during aging. On the other hand, the Raman spectra of all samples showed only the degradation of the bands of the dyes and no new bands of potential degradation products. Therefore, such conversions of the fluorescent dyes to other fluorescent species seem to occur only to a small extent. Quantitative conclusions from the fluorescence spectra, which cannot be drawn from this study because of the conditions of the experiment, would help to interpret these results. Currently, further research is being conducted on the degradation products of the dyes.

## Conclusions

Despite their high light sensitivity, daylight fluorescent pigments are widely used in contemporary art and have been popular with artists since their launch to the present day. The study examined the possible changes of these pigments in exposure to VIS and UV. The aging process was investigated by photography, color measurements, Raman spectroscopy and fluorescence spectroscopy. In this way, the study illustrates the different states of aging of the colors and facilitates the interpretation of the current state of these pigments on art and cultural objects.

The colorimetric documentation of the aging allowed an elaboration of general trends in the aging of the daylight fluorescent pigments from Kremer Pigmente. The positive hue change due to the fading as well as the change of the initial fluorescence color to a more bluish fluorescence was observed for all colors.

In this study, the color changes could be convincingly represented with DL photography as well as UVF photography. Photographs are still an important method of documenting such color changes, especially for art and cultural objects, providing precise photographic documentation with consistent equipment and settings.

Raman spectroscopy has proven to be a suitable method to track the degradation of the main dyes in the daylight fluorescent pigments in VIS aging and the degradation of the optical brighteners in UV aging, which explains the results obtained with the other methods applied. Dyes in very low concentrations and the degradation products could unfortunately not be detected in the Raman spectra due to the complexity of the samples. Fluorescence spectroscopy additionally revealed that the color changes were probably due not only to the decrease in the concentration of the initial dyes, but also to influences from the degradation products. An attempt to clarify the nature of the degradation products using HPLC–MS is currently being performed.

The study allows conclusions to be drawn about the handling of daylight fluorescent paints in conservation practice. The presentation of such artworks should always be well thought out and undertaken with caution regarding light exposure.

## Supplementary Information

**Additional file 1**. Additional figures.

## Data Availability

The data generated or analyzed during this study are either included in this published article and its supplementary information files or are available from the corresponding author on reasonable request.

## Abbreviations

MSF resin: Melamine toluenesulfonamide formaldehyde resin
TLC: Thin-layer chromatography
UV: Ultraviolet radiation
VIS: Visible light
DL photography: Daylight/visible light photography
UVF photography: UV-induced visible fluorescence photography
FRET: Förster resonance energy transfer

## Footnotes

1 1 Mlxh is 1 million lux hours; 1 lux hour (lxh) is the unit of exposure, which is the product of illuminance (lx) and illumination time (h).

2 Further reading: Saunders D. Museum Lighting: A Guide for Conservators and Curators. Los Angeles: The Getty Conservation Institute; 2020.

3 https://www.uvinnovations.com/.
