# Supplementary material for: The light aging behavior of daylight fluorescent paints: a colorimetric, photographic, Raman spectroscopic and fluorescence spectroscopic study
Source: Herit Sci. 2022 Oct 27;10(1):171. doi: 10.1186/s40494-022-00812-4 (PMC9610339; doi:10.1186/s40494-022-00812-4)
Supplement: Supplementary file 1 — Additional file 1. Additional figures. [file 40494_2022_812_MOESM1_ESM.pdf]

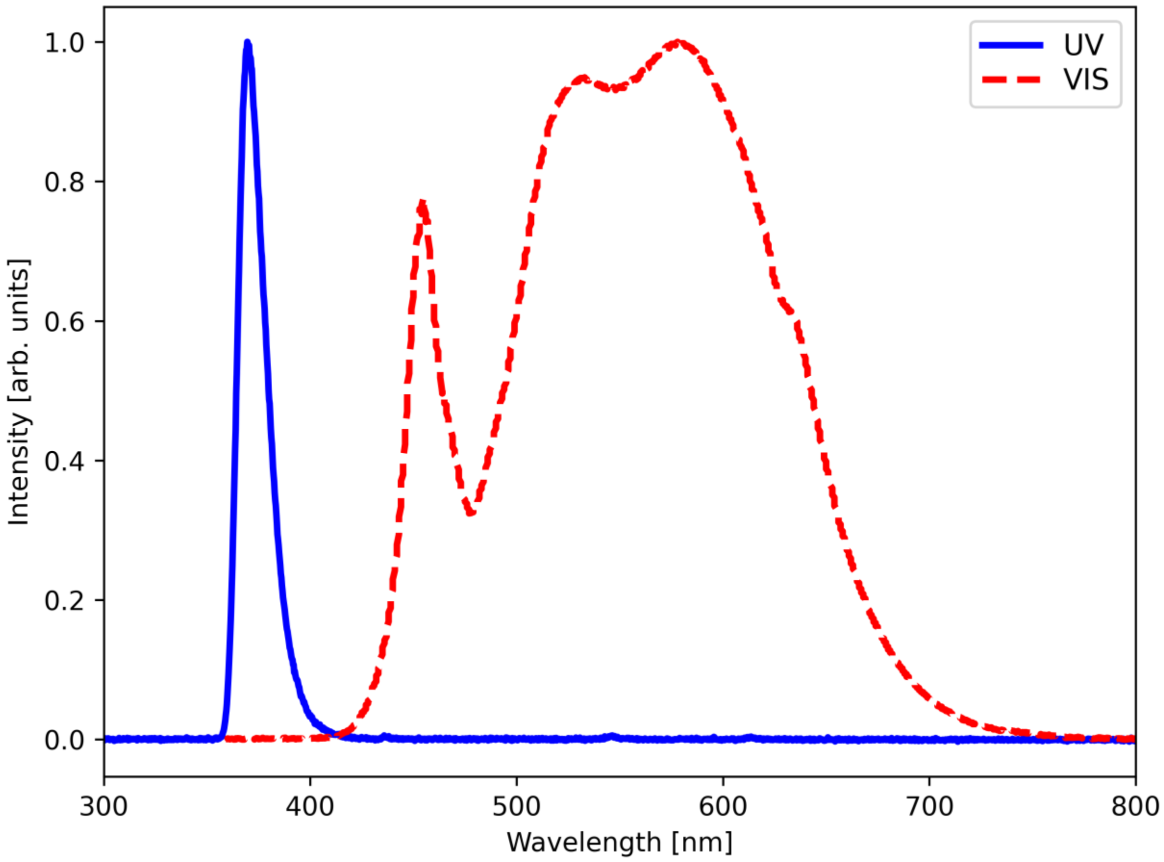

Figure S1: Normalized Intensity of the LEDs used for VIS aging and UV aging. The actual intensity of the UV LEDs is much smaller than that of the VIS LEDs.

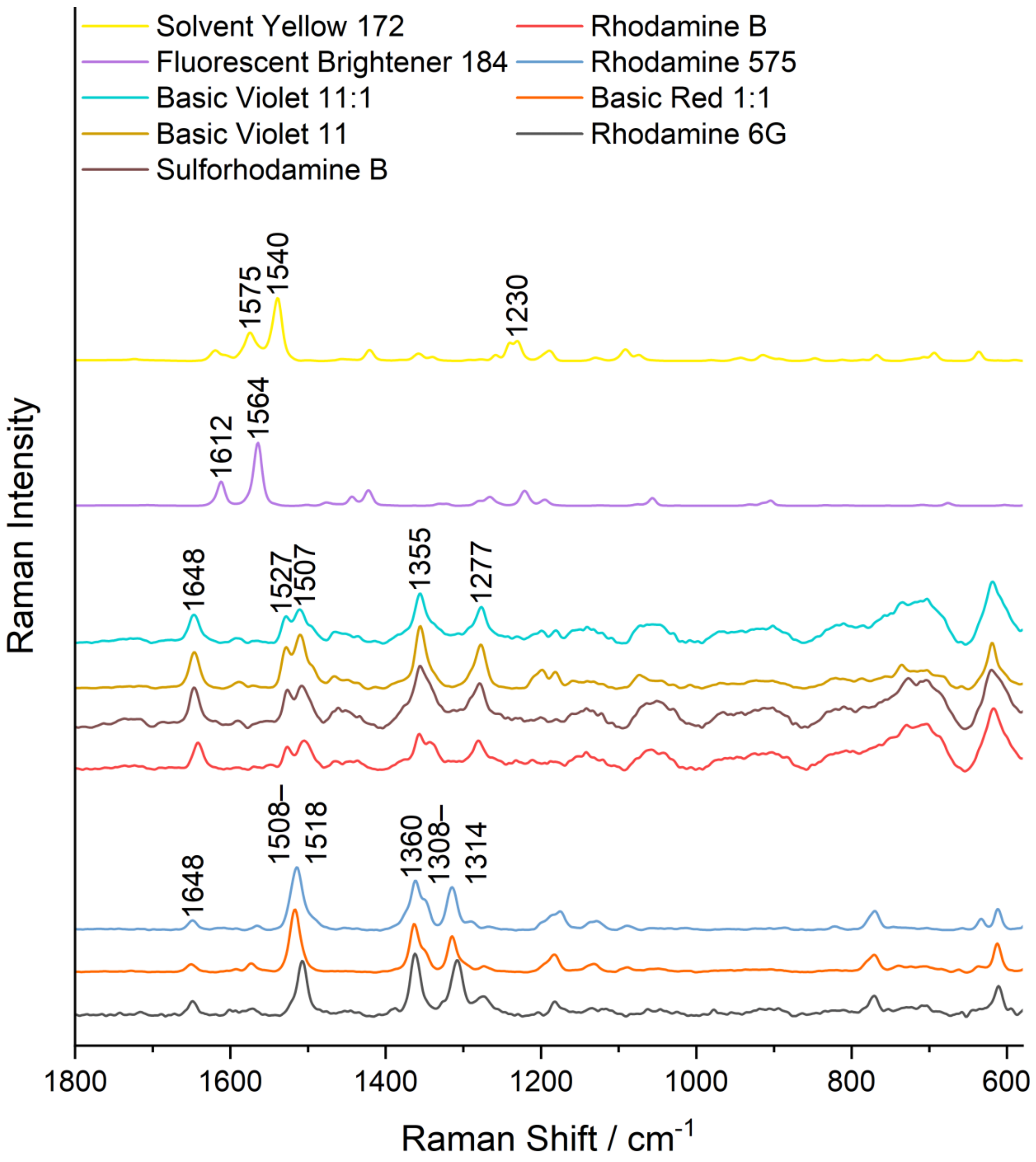

Figure S2: Baseline corrected and normalized Raman spectra of the reference dyes.

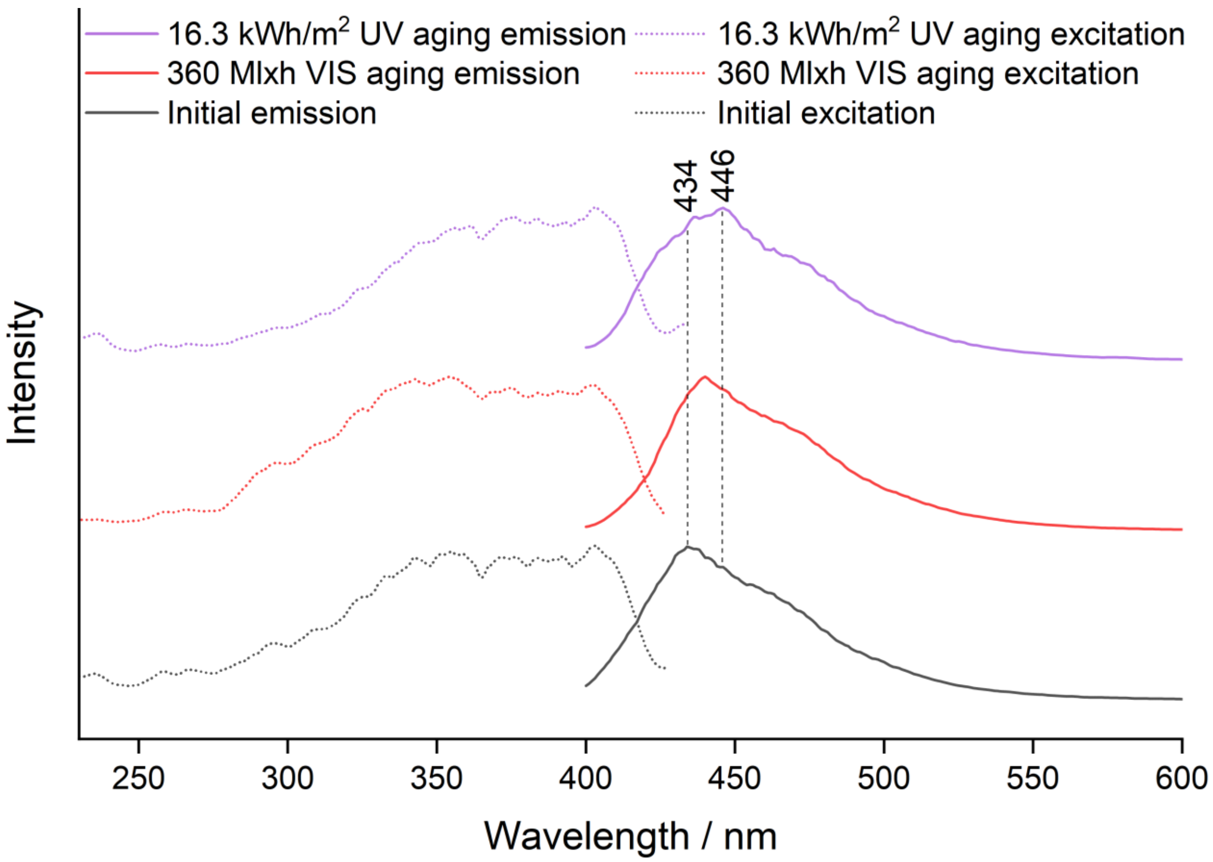

Figure S3: Fluorescence spectra of the White sample before aging, after 360 Mlxh VIS aging and after 16.3 kWh/m² UV aging (from bottom to top).

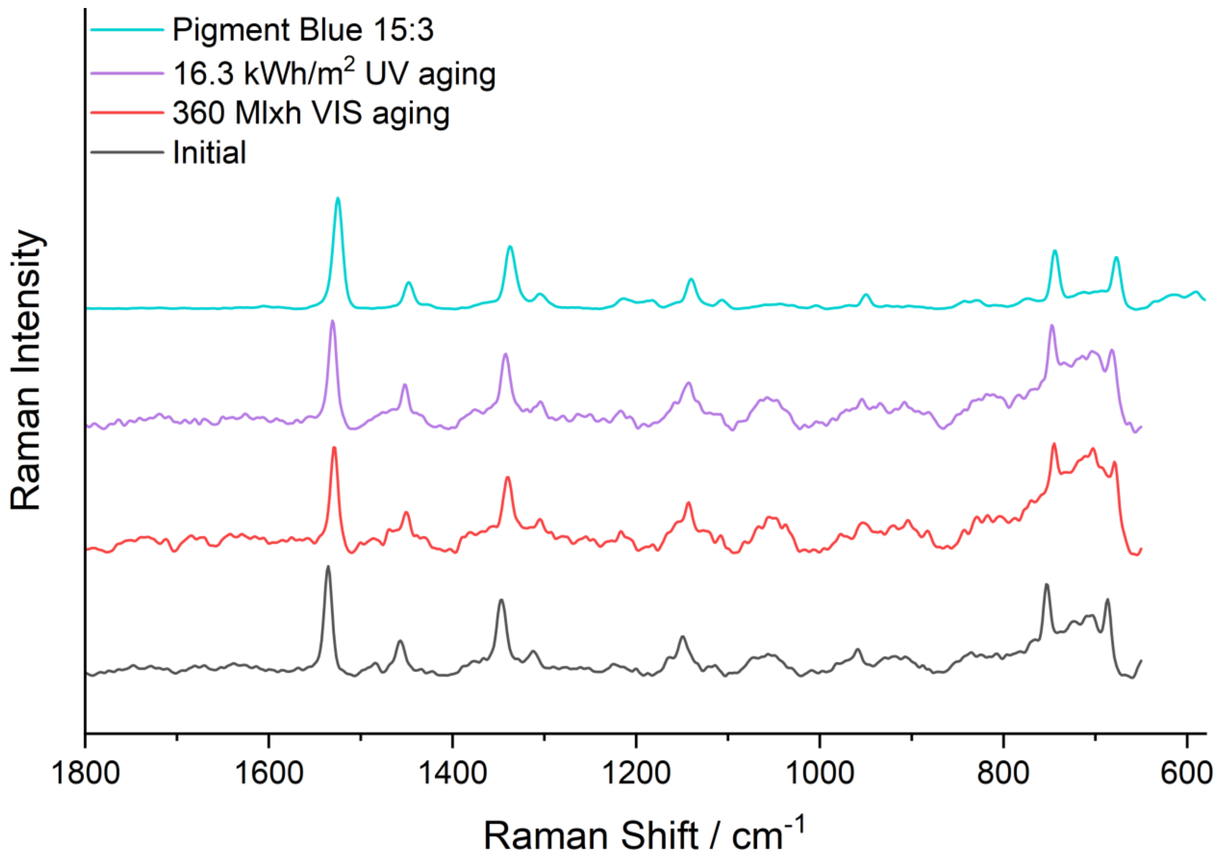

Figure S4: Raman spectra of the Blue sample before aging, after 360 Mlxh VIS aging and after 16.3 kWh/m² UV aging and Raman spectrum of Pigment Blue 15:3 (from bottom to top).

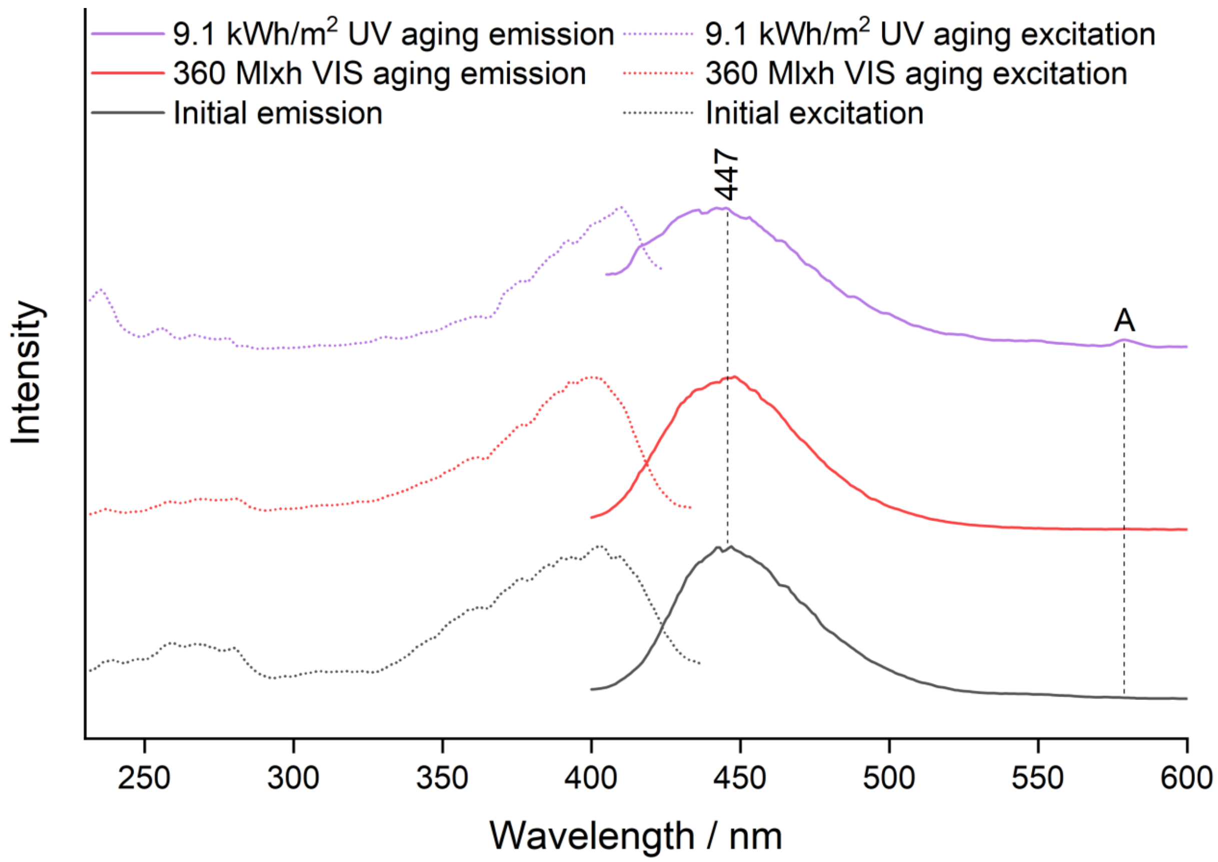

Figure S5: Fluorescence spectra of the Blue sample before aging, after 360 Mlxh VIS aging and after 9.1 kWh/m² UV aging (from bottom to top); **A** – Plextol D498.

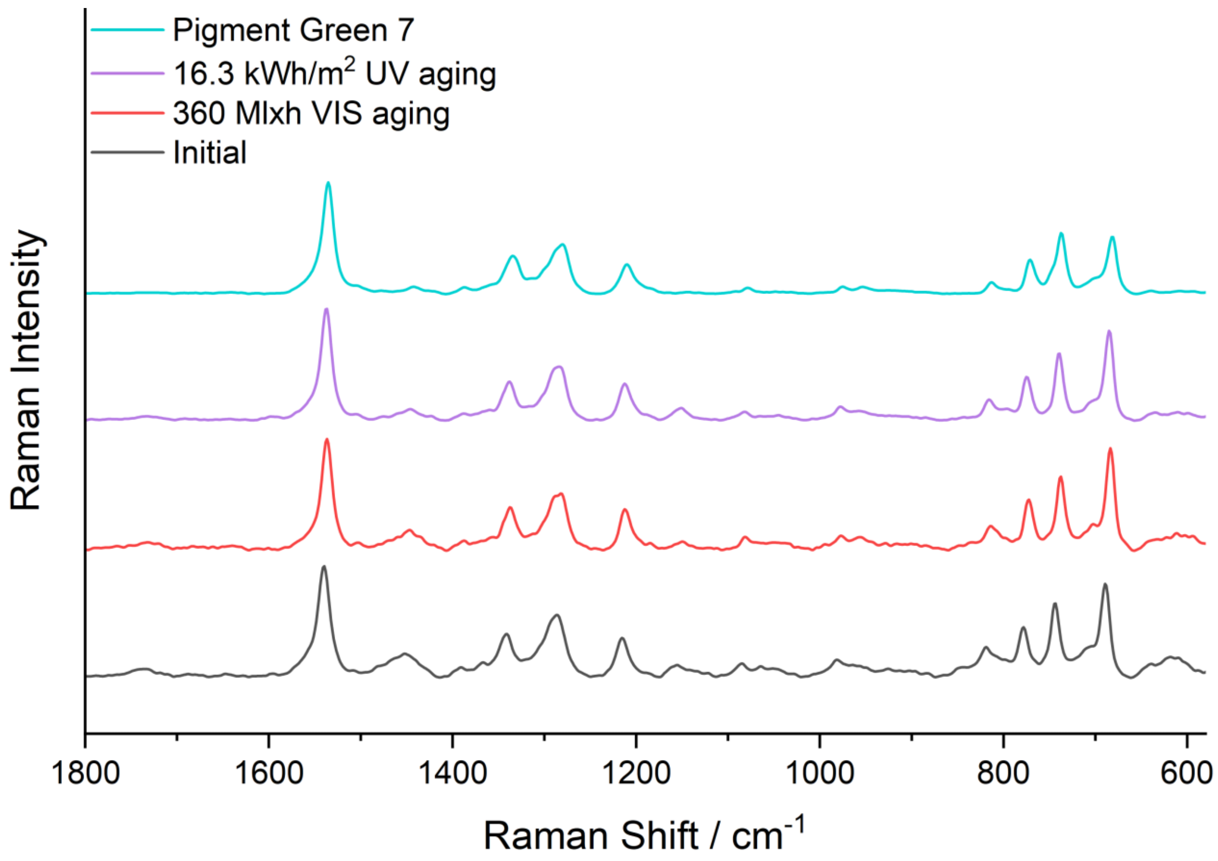

Figure S6: Raman spectra of the Green sample before aging, after 360 Mlxh VIS aging and after 16.3 kWh/m² UV aging and Raman spectrum of Pigment Green 7 (from bottom to top).

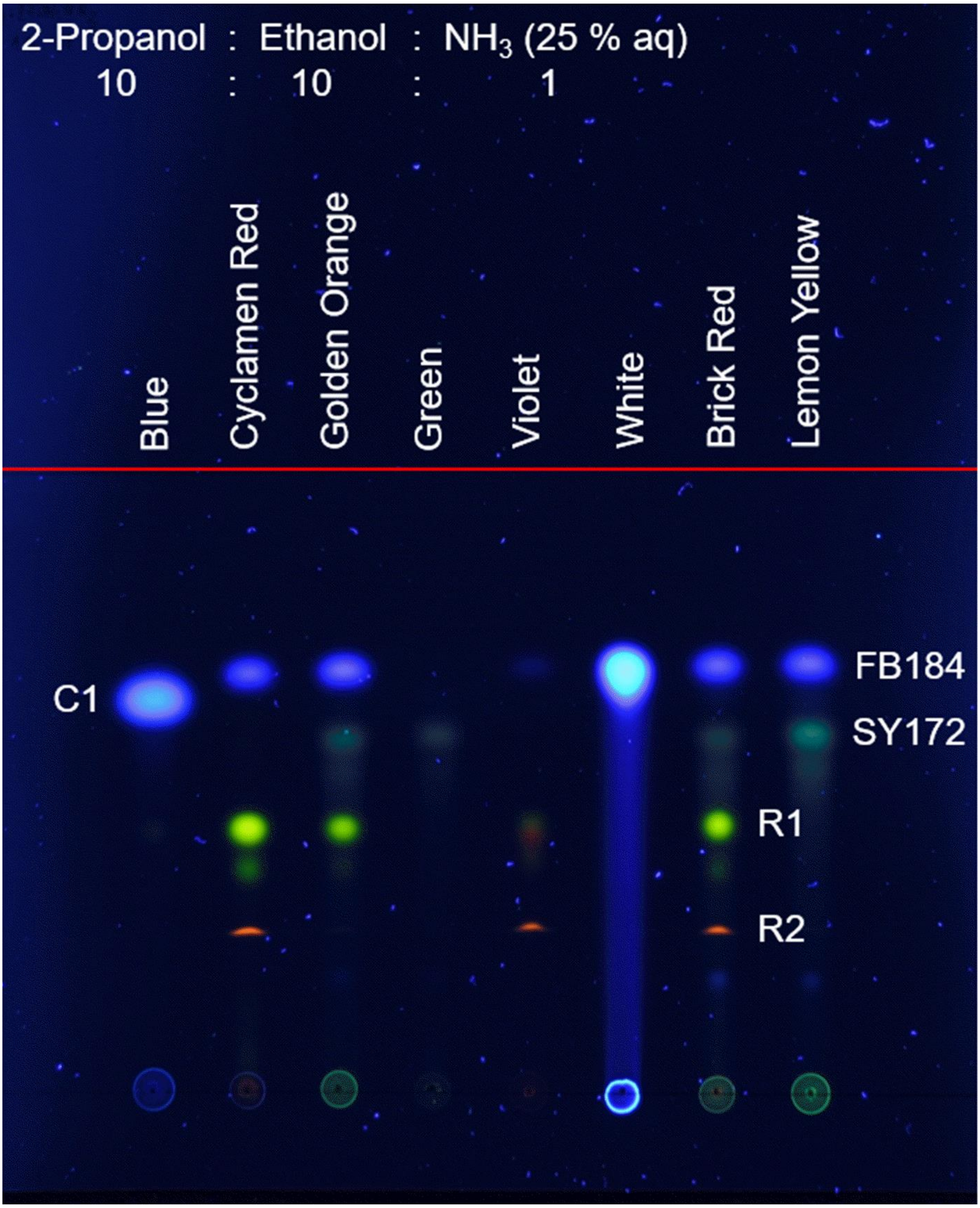

Figure S7: TLC plate of the acetone extracts of the daylight fluorescent pigments from Kremer Pigmente; **C1** – Coumarin 1, **FB184** – Fluorescent Brightener 184, **SY172** – Solvent Yellow 172, **R1** – Group 1 rhodamines, **R2** – Group 2 rhodamines.

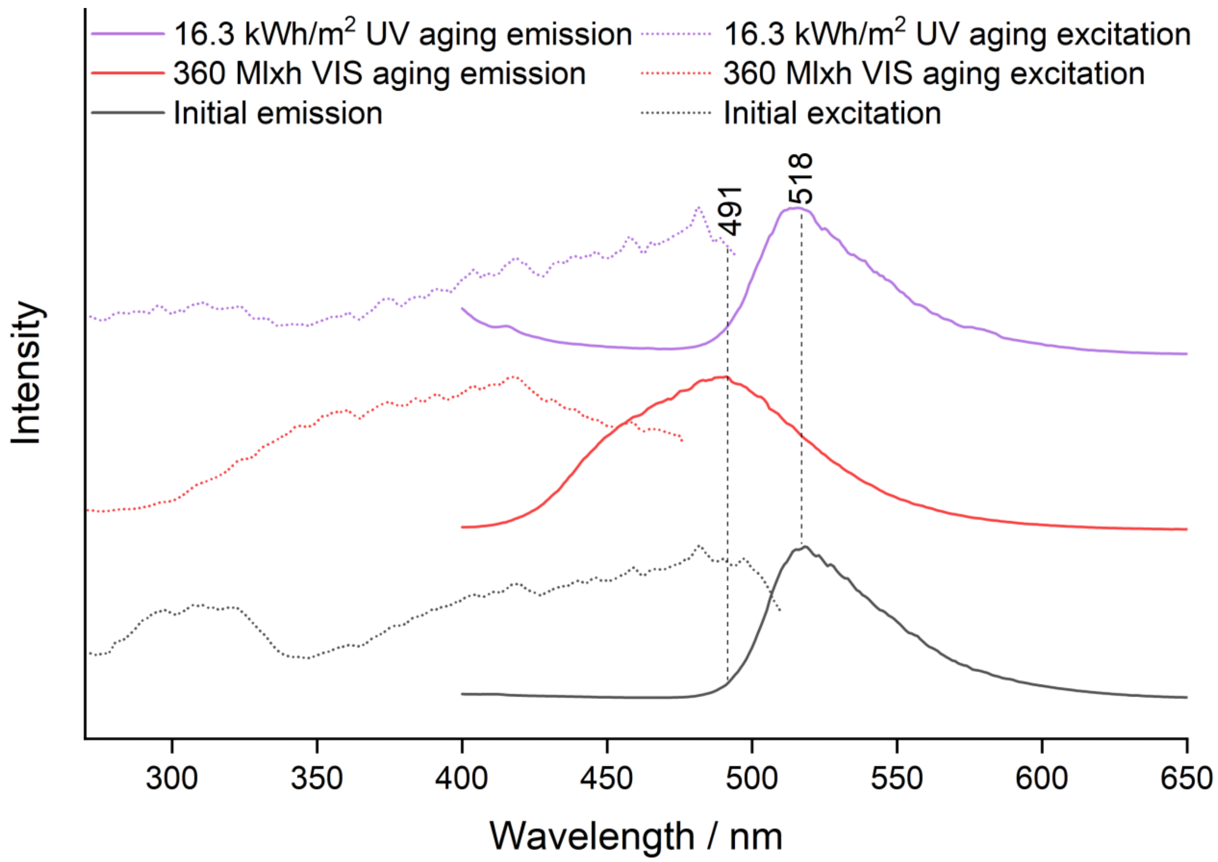

Figure S8: Fluorescence spectra of the Lemon Yellow sample before aging, after 360 Mlxh VIS aging and after 16.3 kWh/m² UV aging (from bottom to top).

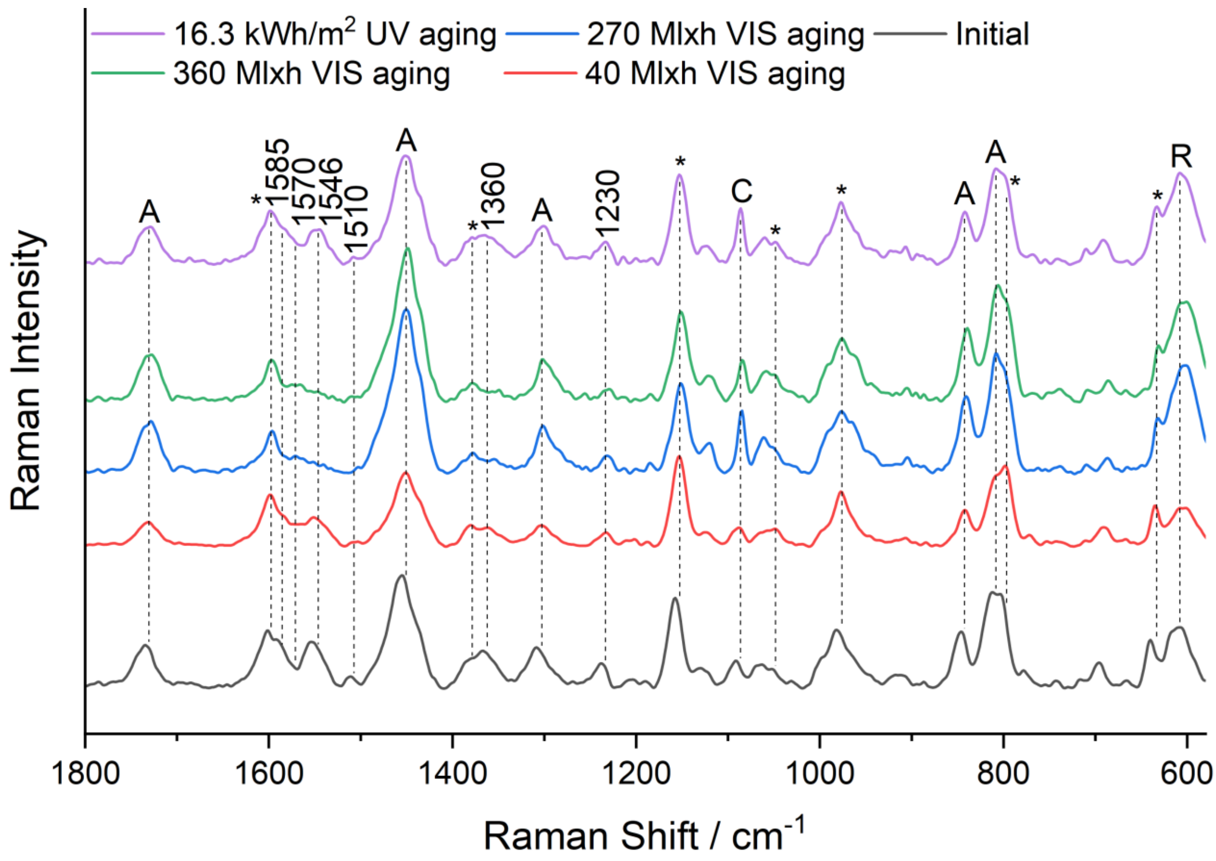

Figure S9: Raman spectra of the Golden Orange sample before aging, after 40 Mlxh, 270 Mlxh and 360 Mlxh VIS aging and after 16.3 kWh/m² UV aging (from bottom to top); **A** – Plextol D498, **R** – Rutile, **C** – Calcite, ***** – MSF resin.

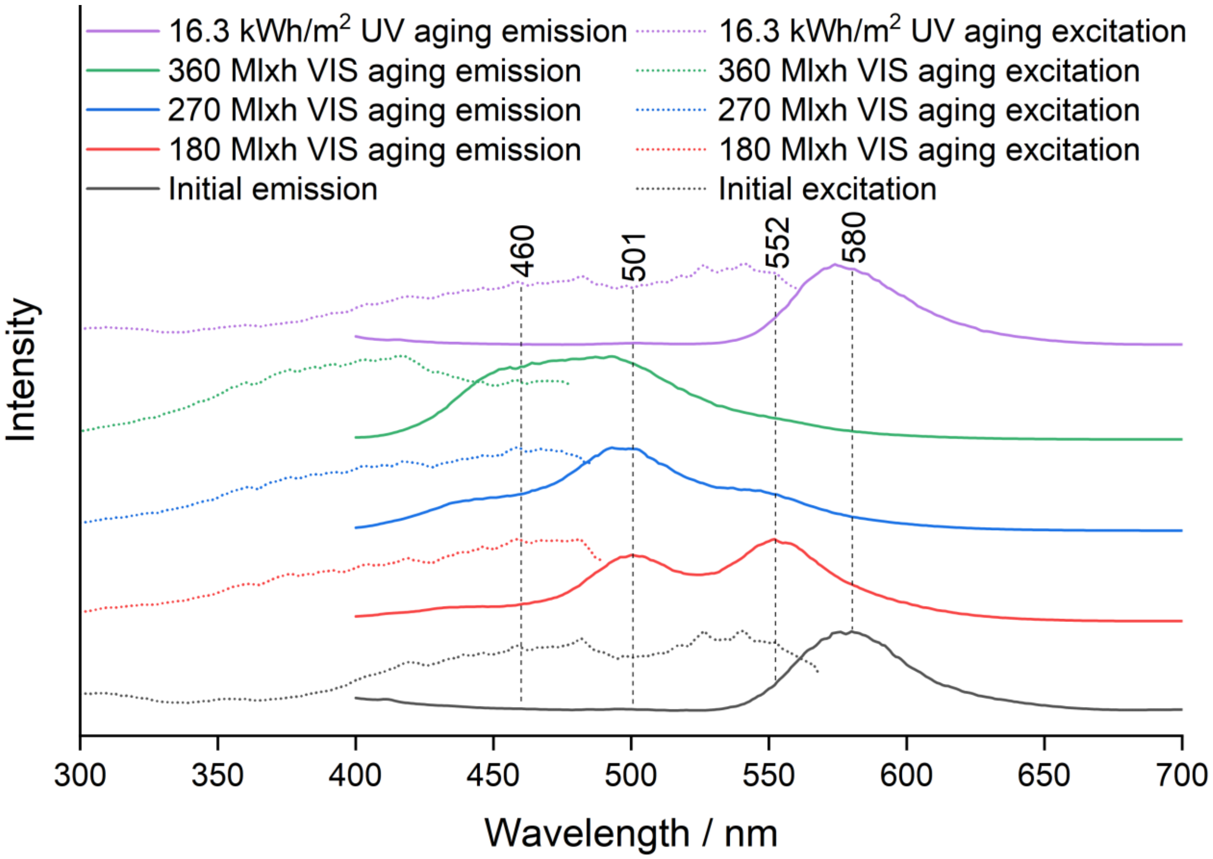

Figure S10: Fluorescence spectra of the Golden Orange sample before aging, after 180 Mlxh, 270 Mlxh and 360 Mlxh VIS aging and after 16.3 kWh/m² UV aging (from bottom to top).

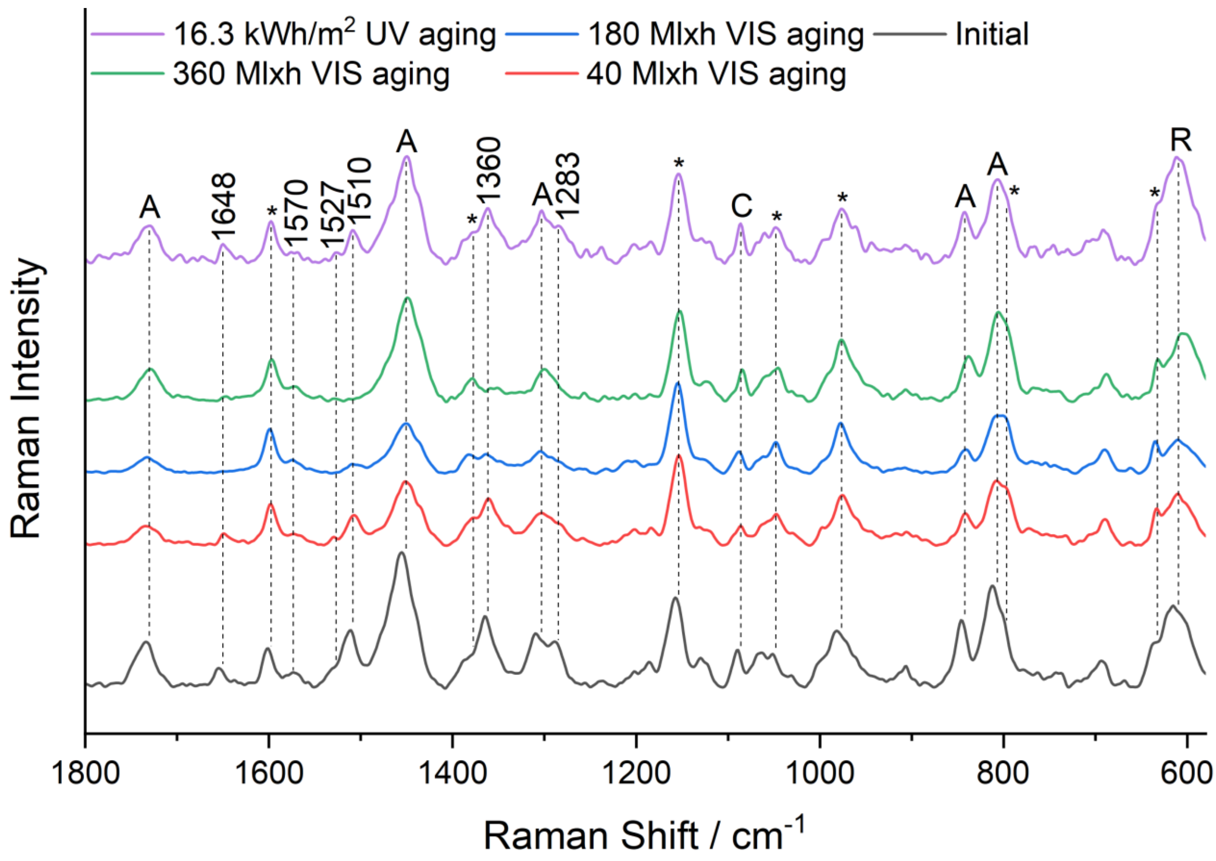

Figure S11: Raman spectra of the Cyclamen Red sample before aging, after 40 Mlxh, 180 Mlxh and 360 Mlxh VIS aging and after 16.3 kWh/m² UV aging (from bottom to top); **A** – Plextol D498, **R** – Rutile, **C** – Calcite, * – MSF resin.

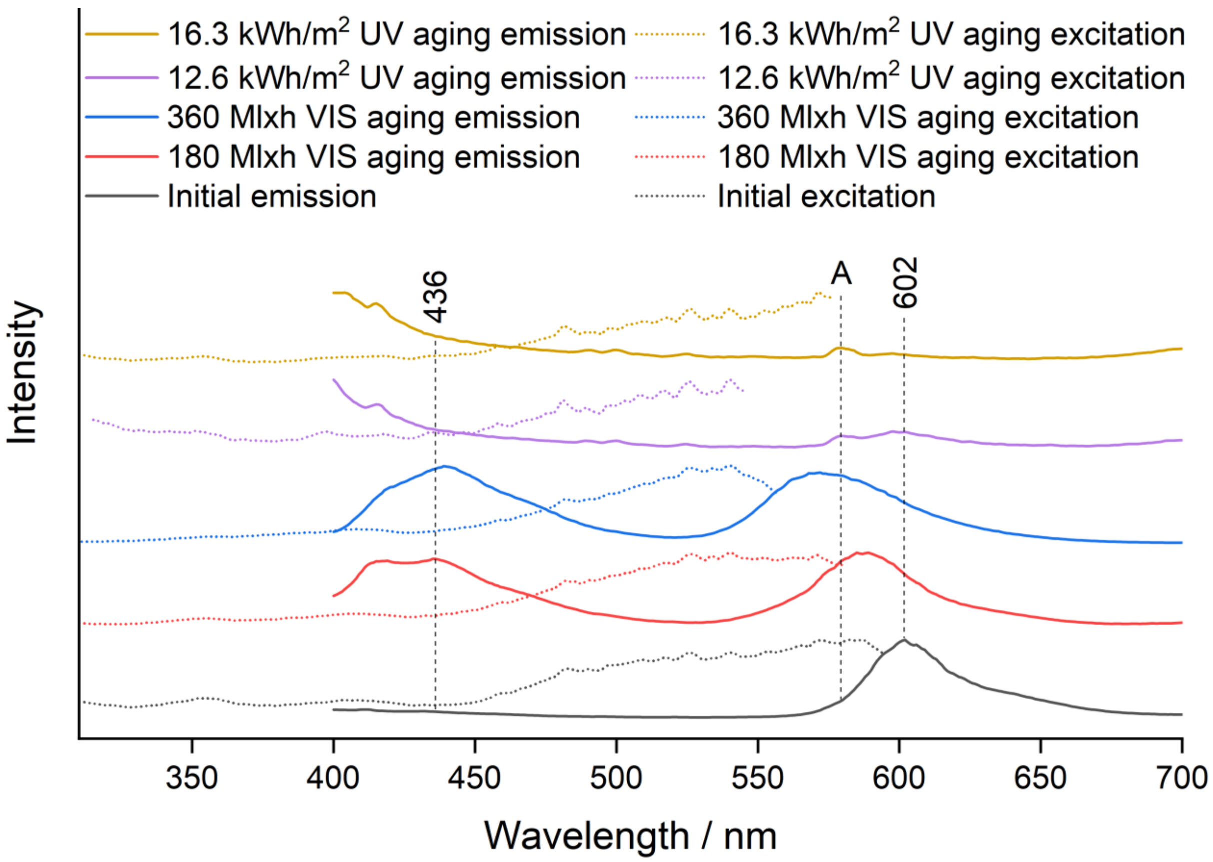

Figure S12: Fluorescence spectra of the Cyclamen Red sample before aging, after 180 Mlxh and 360 Mlxh VIS aging and after 12.6 kWh/m² and 16.3 kWh/m² UV aging (from bottom to top); **A** – Plextol D498.
